# Neurotherapeutic effects of *Ginkgo biloba* extract and its terpene trilactone, ginkgolide B, on sciatic crush injury model: A new evidence

**DOI:** 10.1371/journal.pone.0226626

**Published:** 2019-12-26

**Authors:** Dalal G. Al-Adwani, Waleed M. Renno, Khaled Y. Orabi

**Affiliations:** 1 Department of Pharmaceutical Chemistry, Faculty of Pharmacy, Kuwait University, Safat 13110, Kuwait; 2 Department of Anatomy, Faculty of Medicine, Kuwait University, Safat 13110, Kuwait; Southern Methodist University, UNITED STATES

## Abstract

*Ginkgo biloba leaves extract* (GBE) was subjected to neuroprotective-guided fractionation to produce eleven fractions with different polarities and constituents. The intermediate polar fraction was shown to be terpene trilactones-enriched fraction (TEGBE). Out of this fraction, pure ginkgolide B (G-B) was further purified and identified based on its spectral data. The effects of GBE and TEGBE were evaluated in comparison to that of G-B in the crush sciatic nerve injury rat model. To evaluate the neuroprotective effects, sixty Wistar male rats were randomly allocated into 6 groups: naive, sham, crush + normal saline, and three treatment groups; crush + GBE, crush + TEGBE, and crush + G-B. Treatments were given one hour following injury, and once daily for 14 days. Neurobehavioral tests, histomorphological examinations, and immunohistochemical analysis of the sciatic nerve and the spinal cord were performed at weeks 3 and 6 post-injury. GBE, TEGBE and G-B were shown to enhance the functional and sensory behavioral parameters and to protect the histological and the ultrastructural elements in the sciatic nerve. Additionally, all treatments prevented spinal cord neurons from further deterioration. It was shown that G-B has the most significant potential effects among all treatments with values that were nearly comparable to those of sham and naive groups.

## Introduction

**Fig 1:**
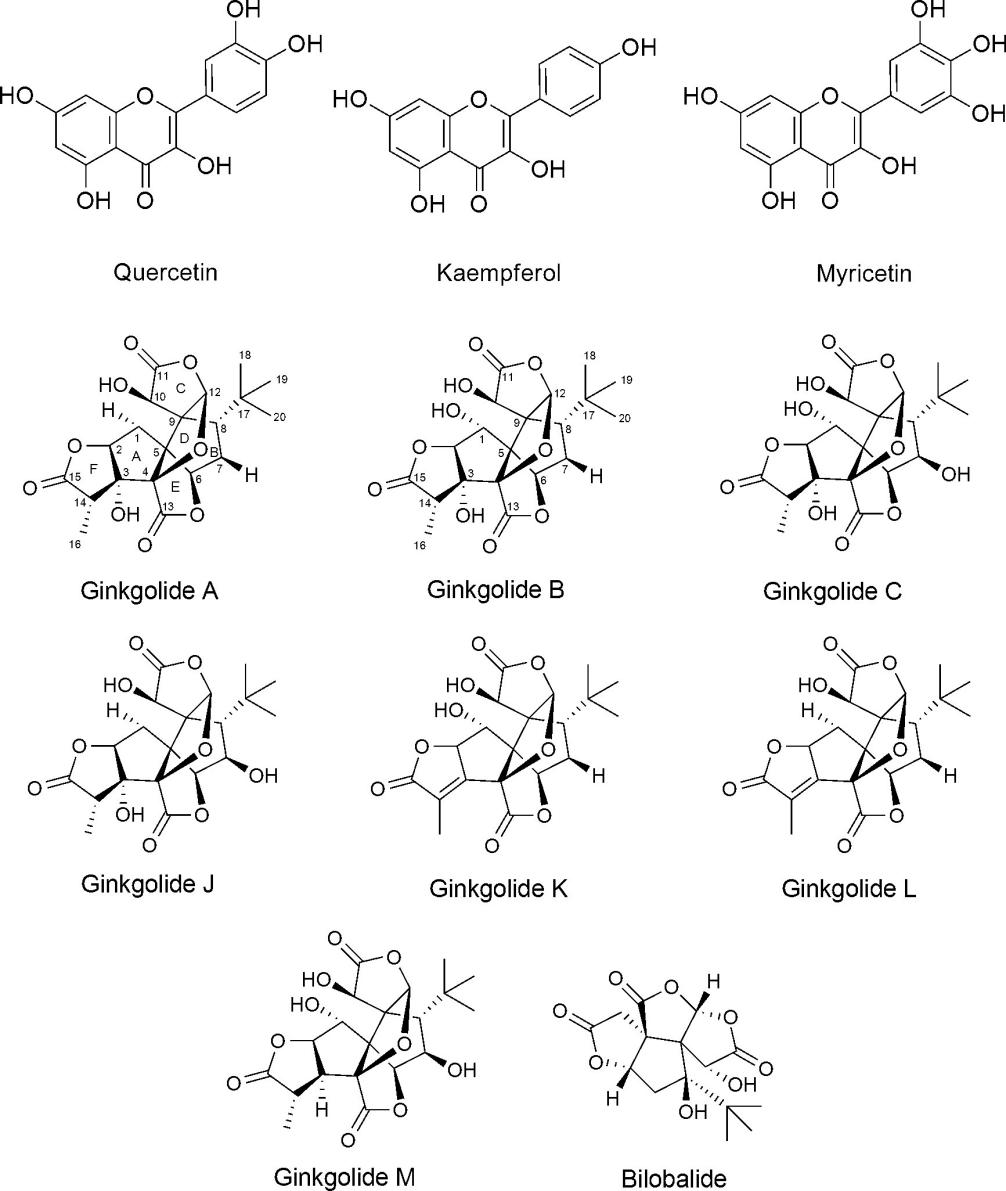
Common flavonols and terpene trilactones in *Ginkgo biloba* leaves.

*Ginkgo biloba* L. belongs to the Family Ginkgoaceae and is reported to have a wide range of biological activities due to the synergistic effects of its active components, mainly the flavonols, e.g., quercetin, kaempferol and myricetin, and terpene trilactones (Fig 1).

Typical flavonoids concentrations, in the standardized extracts, range from 24–26%, and the trilactones from 6–8% [1]. Thus, low quality extracts may be subjected to adulteration with some of these flavonoids to meet the minimum requirements of the flavonoids content.

*In vitro* and *in vivo* studies showed that the chemopreventive effects of these flavonoids are due to their anti-proliferative, apoptosis, cytotoxic, and angiogenesis actions in cancer models [2–5]. Some researchers have reported the role of flavonoids in preventing the development of Alzheimer’s disease due to their capabilities to attenuate the oxidative stress in the patients [6]. Consequently, flavonoids are considered neuroprotective agents and contribute to the total effects of *Ginkgo biloba* on cerebrovascular and peripheral nervous system.

Additionally, terpene trilactones, unique components of *Ginkgo biloba*, have received, by far, the most attention among other compounds in *Ginkgo biloba*, due to their activity and, thus, importance in the standardization process of GBE. Terpene trilactones are divided structurally into two groups; diterpene trilactones, e.g., ginkgolides A, B and C, and sesquiterpene trilactones, i.e., bilobalides (Fig 1).

Ginkgolides were found to be selective antagonists of platelet activating factor (PAF) receptors [7] with ginkgolide B (G-B) to be the most potent [8]. It was documented that G-B increases the ischemic reperfusion blood flow following cerebral injury [9].

The affinity to PAF receptors was concluded to be due to the cage-like structure of ginkgolides. It is interesting to notice that the presence of an hydroxyl group at C-7 converts ginkgolide B into C, and consequently lowers the activity [10].

Moreover, it was reported that ginkgolide C exhibits the highest affinity to alpha-1 glycine receptors, and that modification of any of the hydroxyl groups leads to the loss of activity [11]. Consequently, the presence of hydroxyl groups in ginkgolides revealed a crucial role in their activity, and any chemical modulation may have a critical impact on their affinity to the target. This is why different ginkgolides, differ only in these hydroxyl groups, have different level of activities.

Bilobalide, on the other hand, is considered to be the most potent neuroprotective compound among other trilactones. Such an effect is due to the strong inhibition of phospholipase A2, the activation of which initiates a cascade of events leading to neuronal death [12]. In addition, bilobalide was reported to antagonize the GABA_A_ receptor in rat hippocampal tissue, which is also contributing to the neuroprotective effect of bilobalide [13].

Peripheral nerve injury presents a lifelong disability where crush injury is the highest rated type among other injuries [14]. Sciatic nerve crush injury underlies a serious problem with an incidence of 2.8% in multiple-trauma victims and is diagnosed as allodynia, and heat and mechanical hyperalgesia [15]. Among other therapeutic strategies, pharmacotherapy has been shown to be a promising approach to the neurorehabilitation, with the exploration of potential leads from nature being of increasing interest [15].

Towards that, many reports discussed the role of GBE in nerve injury and peripheral neuropathic pain [16–18]. However, the neurotherapeutic effect of GBE on the peripheral nerve crush injury in rats has been rarely studied, particularly on the sciatic nerve.

This study aimed at developing a neuroprotective-guided fractionation protocol to possibly isolate and identify the active leads(s) from GBE. GBE was prepared, then fractionated to afford eleven fractions with different polarities. Fractions containing mainly ginkgolides (TEGBE) were further processed to isolate a representative ginkgolide, i.e., ginkgolide B (G-B). The potential neurotherapeutic effects of GBE, TEGBE and G-B were evaluated on the crushed sciatic nerve. Several tests such as neurobehavioral, histomorphological and immunohistochemical analyses of the sciatic nerve and spinal cord were done.

## Materials and methods

### Equipment and chemicals

UV spectra were measured in methanol and recorded using an UV-Visible dual beam-spectrophotometer (Spectroscan 50), while IR spectra were recorded on a JASCO FT/IR-4200 spectrophotometer. ^1^H and ^13^C NMR spectra of G-B were obtained using a Bruker Avance II 600 MHz spectrometer. Both the ^1^H and ^13^C spectra were recorded in deuterated methanol (MeOH-*d*_*4*_)_,_ and the chemical shift values were expressed in parts per million (ppm) relative to the internal standard, tetramethylsilane. Carbon multiplicities were determined using DEPT angles at 90°, 45°, and 135°. Two-dimensional NMR data were obtained using the standard pulse sequence of the Bruker 600 for correlation spectroscopy (COSY), heteronuclear single quantum coherence (HSQC) and heteronuclear multiple bond correlation (HMBC). HREIMS data were determined using a double-focusing magnetic sector mass spectrometer (GS-MS DFS/Thermo). High pressure liquid chromatography (HPLC) analyses were performed using Waters machine equipped with a reversed-phase column (ODS, 5 μm, 4.6 x 150 mm, Waters), and Photodiode Array Detector (PDA, Waters). Additionally, preparative HPLC separations were done on Waters HPLC machine equipped with XBridge^TM^ Prep column (ODS, 5 μm, 10 x 150 mm, Waters). The operating module was equipped with Empower software. Solvents used for chromatographic fractionation and thin layer chromatographic (TLC) analyses were of a general-purpose reagent (GPR) grade, and those for HPLC and spectral analyses were of HPLC and analytical grades, respectively.

### Plant material

Coarsely powdered *Ginkgo biloba* leaves were purchased from Vitaspace, USA. Pure authentic ginkgolides A, B, C and bilobalide were obtained commercially (Merck, Germany) to serve as reference standards.

### Extraction

Three kilograms of the powdered *Ginkgo biloba* leaves were percolated in 10 l of 80% methanol for 24 hours. The extract was collected and the percolation process was repeated two more times using fresh methanol each time. The combined methanol extracts were evaporated *in vacuo* till dryness to afford 70 g of brownish syrupy residue, abbreviated as GBE.

### Neuroprotective-guided fractionation

Bioactivity-guided fractionation protocol was followed, where 50 g of GBE were fractionated using vacuum liquid chromatography [19]. Initially, the extract was pre-adsorbed on a 50 g C18 silica gel. The loaded silica was applied on a 400 g C18 silica gel column (8 x 30 cm ODS, 40–63 μm, 230–400 mesh, ASTM, Merck). The column was initially eluted with 100% water. The elution was run in a gradient mode, where the eluent strength increased by 10% increments of methanol in water, and ended up with 100% methanol. One-liter fractions of each polarity were collected, evaporated and weighed. This afforded 11 fractions, DGA-I-5A – 5K, with different polarities. TLC analyses of the obtained fractions were carried out using pre-coated glass plates (5 x10 cm, 250 μm) with UV_254_ indicator (Anal Tech). The plates were developed in toluene: acetone; 8:2, then, visualized *via* spraying with acetic anhydride spray reagent, heating for 10 minutes, and then, exposing them to both long (λ_max_ = 366 nm) and short (λ_max_ = 254 nm) wavelengths of the UV light (CAMAG).

#### HPLC analysis

**Fig 2:**
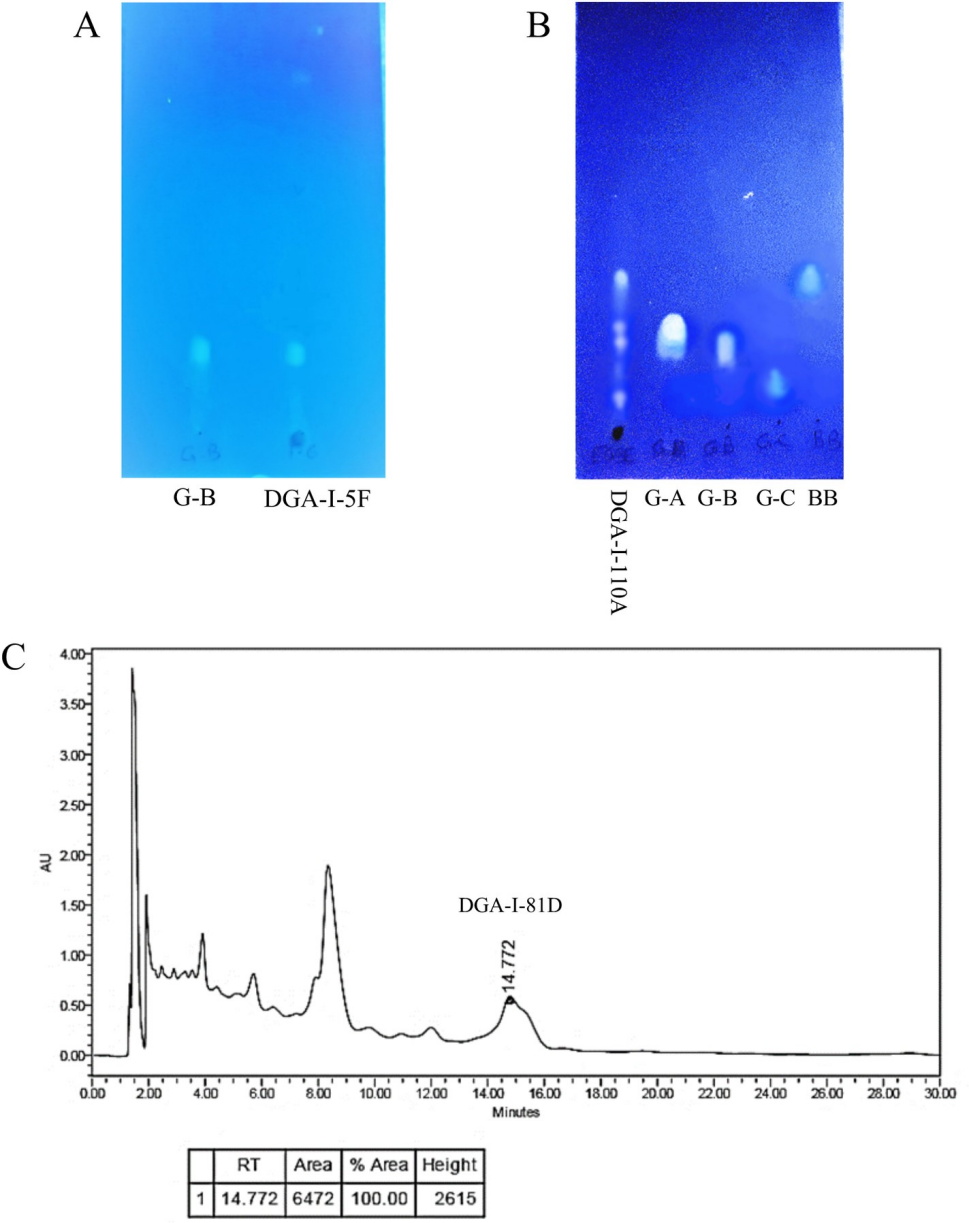
Chromatographic analyses of some *Ginkgo biloba* fractions. **A:** TLC analysis of fraction DGA-I-5F along with authentic ginkgolide B (G-B). TLC plate was developed in toluene: acetone; 8:2, and visualized via exposure to long wavelength UV light (λ_max_ = 366 nm). **B:** TLC analysis of terpene trilactones-enriched extract (TEGBE) (DGA-I-110A) compared to authentic terpene trilactones references (toluene: acetone 8:2, UV light λ_max_ = 366 nm). **C:** Preparative HPLC chromatogram for fraction DGA-I-5F. Peak 1 (retention time = 14.7 min) was isolated and purified. This compound was abbreviated DGA-I-81D and was identified as ginkgolide B.

A modified HPLC method from a previously reported one [20] was proposed and applied to analyze, isolate and purify G-B. Fractions DGA-I-5F and 5G, eluted by 50% and 60% methanol in water, respectively, and shown to contain G-B (TLC analysis, Fig 2A and 2B), were subjected to HPLC analysis for further confirmation (Fig 2C).

On the other hand, preparative HPLC was used to isolate G-B in quantities large enough to evaluate its pharmacological effects. Authentic G-B sample (10 mg dissolved in 1 ml of MeOH) was initially analyzed to find the reference sample’s retention time and the wavelength corresponding to the maximum absorbance. Then, 8 mg of each of the above two fractions were dissolved in 2 ml of MeOH, filtered and subjected to analysis. Authentic G-B and the two fractions were injected in a volume of 20 μl each, and eluted using methanol: tetrahydrofuran: water; 1: 3: 15, as the mobile phase, at a flow rate of 1.5 μl/min for a total elution time of 25 minutes. Detection was done using a PDA detector set at λ_max_ = 219 nm. The produced chromatograms from authentic G-B were compared to those obtained from the other two fractions.

#### Isolation and purification of G-B

Further purification processes were performed to isolate G-B using preparative HPLC. The solvent system used in the analytical HPLC method (MeOH: THF: H_2_O; 1: 3: 15) was applied in the preparative HPLC protocol. The reference compound, G-B, was initially tested, following a dilution of 10 mg in 500 μl MeOH, to confirm the running instrument parameters, to use the obtained chromatogram as a reference for collecting the pure isolated sample. Reference G-B was injected at a volume of 100 μl, and eluted at a flow rate of 5 ml/min for a 25-minute duration. The chromatogram of G-B was recorded. Fractions DGA-I-5F and 5G were diluted appropriately in MeOH (1 g in 10 ml), and filtered as before. The filtered fractions were injected at a volume of 500 μl and a flow rate of 5 ml/min for a total run time of 30 minutes. Different peaks were separately collected, as detected by the PDA detector (λ_max_ = 219 nm). This procedure was repeated several times, and similar fractions were collected and finally pooled together. The pooled fractions were dried, and tested by TLC to confirm the presence of pure G-B, abbreviated as DGA-I-81D.

**DGA-I-81D:** amorphous powder (0.263 yield %); UV (MeOH) λ_max_ (log ε) 218 (3.45) nm; IR (neat) *v*_max_ 3446 and 1774 cm^-1^; ^1^H NMR (CD_3_OD, 600 MHz) see Table 1; ^13^C NMR (CD_3_OD, 150 MHz) see Table 1; HREIMS (70 eV) *m/z* 424.1365 [M]^+^; R_*f*_ = 0.19 (toluene: acetone; 8:2).

**Table 1:** ^1^H (600 MHz), ^13^C NMR (150 MHz) and HMBC Data (CD_3_OD) of DGA-I-81D.

position	δ_c_, multiplicity	δ_H_ (*J* in Hz)	HMBC^*a*^	
			^3^*J*	^2^*J*
1	75.6, CH	4.20, d (7.8)	6, 9	2
2	93.5, CH	4.59, d (7.8)	5, 15	1, 3
3	84.8, C	—	—	—
4	100.3, C	—	—	—
5	73.7, C	—	—	—
6	80.9, CH	5.41, d (4.2)	1, 8, 9, 13	—
7	38.3, CH_2_	H_α_: 2.11, ddd (13.8, 12.0, 4.2) H_β_: 2.27, dd (13.8, 4.8)	17 5, 9	8 6
8	50.6, CH	1.92, dd (14.4, 4.8)	10, 12, 18, 19, 20	7, 9, 17
9	69.3, C	—	—	—
10	70.8, CH	5.11, s	5, 8	9, 11
11	175.4, C	—	—	—
12	112.1, CH	6.09, s	5, 8, 11	9
13	172.8, C	—	—	—
14	43.5, CH	3.03, q (7.2)	4	3, 15, 16
15	178.5, C	—	—	—
16	8.2, CH_3_	1.24, d (6.6)	3, 15	14
17	33.5, C	—	—	—
18	29.6, CH_3_	1.13, s	8	17
19	29.6, CH_3_	1.13, s	8	17
20	29.6, CH_3_	1.13, s	8	17

^*a*^ HMBC correlations, optimized for 8 Hz, are from proton(s) stated to the indicated carbon.

#### Terpene trilactones-enriched fraction (TEGBE)

Fractions DGA-I-5H and 5I, eluted by 70% and 80% methanol in water, respectively, in the fractionation step above, were subjected to TLC analysis to verify its content. These fractions were added to the leftover subfractions after eluting G-B from fractions DGA-I-5F and 5G. The pooled together fractions were dried, weighed, and abbreviated TEGBE. This fraction was then subjected to HPLC analysis to confirm the identity of the terpene trilactones.

### Animals

Male Wistar rats, weighing 300–350 g and aging between 2–3 months old, were obtained from the animal facility of the Health Science Center, Kuwait University. The animals were kept under conditions of constant temperature (23±2°C) and humidity with 12/12-hr light/dark cycle. The rats were housed in pairs with food and water ad libitum. Ethical approval was obtained for all procedures from the animal ethics committee at the Health Sciences Center, Kuwait University, Kuwait, and in accordance with the guidelines of laboratory animal welfare and the National Institutes of Health guide for the care and use of Laboratory Animals (NIH Publications No. 8023, revised 1978). All efforts were made to minimize animal suffering and to reduce the number of animals used in the study.

#### Surgical procedures on animals

A total of 60 rats were randomly assigned to 6 groups; naive (no surgery or sciatic nerve injury) (n = 6); sham (sham-injury surgical control group) (n = 12); crush (saline-treated, crushed sciatic nerve) (n = 12); crush + GBE (50 mg/kg GBE-treated crushed sciatic nerve rats) (n = 6); crush + TEGBE (50 mg/kg TEGBE-treated crushed sciatic nerve rats) (n = 12); and crush + G-B (15 mg/kg G-B-treated crushed sciatic nerve rats) (n = 12). All treated groups received i.p. injections of the treatment an hour post-surgery and once daily for 14 days. Doses, route of administration and duration of treatments were established previously in many studies [21]. In this study, the sciatic nerve crush injury was performed as previously described [22]. All approved parameters were followed to minimize animal-animal variation due to injury and to induce a standard direct trauma as previously described [23]. Briefly, animals were anesthetized with an intraperitoneal injection of a mixture of ketamine and xylazine. The sciatic nerve was crushed at a distance of 10 mm from the sciatic notch for 60 seconds using micro mosquito forceps (12.5 cm, straight, World Precision Instruments, Inc.). The nerve was examined to ensure that the epineurial sheath was intact but translucent (axotomy). Then it was replaced under the muscle, and the skin incision was sutured. Sham surgery was done for the rats in the sham group, where the right sciatic nerve was exposed as described before, and skin was sutured, without crushing the nerve [24].

#### Assessment of motor and sensory functional recovery

The rats in all experimental groups were evaluated for motor and sensory neurobehavioral functions 2 weeks preoperatively and 6 weeks post-injury as described before [25]. All tests were repeated three times (with 3 min—20 min interval) for each rat. The mean of the 3 measurements was used as the data point for that rat for calculating the group mean for further statistical analysis. Investigators were blinded to all treatments in all experiments.

Several tests of reflexive sciatic nerve function (motor tests), such as foot position, toe spread, extensor postural thrust (EPT), hopping and rotarod tests were conducted as described in previous studies [22]. Rotarod performance was measured using the rotarod test instrument (47750—Rat Rotarod NG, Italy) set at the acceleration mode. The time and the number of rounds for each rat were recorded once it felt into its lane. The rotarod time latency that the animal falls is indicated on the Y-axis.

Neurobehavioral sensory tests (mechanical and thermal hyperalgesia, and tail flick tests) were evaluated for all experimental animals as previously described [26]. Mechanical hyperalgesia was measured using Analgesia Meter (Ugo-Basile, Linton instrumentation, Italy). This test was used to calculate the percentage of sensory deficits and paw pressure latencies following sciatic nerve injury. The maximum applied force was 250 g to avoid skin damage. Thermal nociception was evaluated using hot/cold plate (50°C; Ugo-Basile, Hot/Cold Plate, Linton instrumentation, Italy). The time between the placement of the animals on the plate and the onset of paw licking, jumping off the plate, and shaking as the paw withdrawal latency was noted. A standard cutoff latency of 35 sec was employed to avoid animal injury. Tail flick test was applied to assess the central nociception using Analgesia Meter Apparatus (Ugo-Basile, Linton instrumentation, Italy).

#### Processing of sciatic nerve tissue for light and electron microscopy

For morphometric analysis and TEM study, half of the animals were randomly selected and sacrificed at week 3 (day 21), and week 6 (day 42) for histopathological and morphometric evaluation as previously described [25]. Specimens of sciatic nerves were cut and processed following the previously published protocol [27]. Briefly, rats were euthanized with carbon dioxide, the right sciatic nerve and lumbar spinal cord were dissected and fixed by immersion overnight at 4° C, in a mixture of 2% glutaraldehyde and 2% paraformaldehyde in 0.1 M phosphate buffer, pH 7.4. On the following day, sciatic nerve specimens distal to the crush sites (or corresponding location in control rats) were washed in phosphate buffer (pH 7.4) twice, post-fixed in 1% osmium tetroxide, dehydrated through a graded alcohol series, immersed into propylene oxide and embedded in Epon resin [25, 27]. From each tissue block, semi-thin (1 μm) cross-sections were cut using RMC MT-7 ultra-microtome (Research and Manufacturing Co, Tucson, AZ, USA), and stained with 1% toluidine blue for light microscopic histopathological examination and morphometric analysis [25, 27]. A light microscope (Olympus BH 40, Tokyo, Japan) equipped with Olympus DP71 digital camera and an image manager system (Olympus, DP-Controller) was used for conducting the histomorphometric analysis on each semi-thin sections of the sciatic nerve. For TEM, ultrathin sections were cut from the same tissue blocks and processed for EM analysis.

#### Morphometric / stereological analysis

It was performed according to the principles described previously [24]. Sections were randomly photographed under a 100x oil-immersion objective via DP-Controller-Olympus software. The number of nerve fibers/field in each photomicrograph were counted. The two fundamental parameters, axon diameter (d) and nerve fiber diameter (D), were measured. Myelin thickness [m = (D-d)/2], g-ratio (d/D) and myelin thickness/axon diameter ratio were calculated manually. The thickness measurements acquired from all sampled axons were then averaged to obtain the mean myelin thickness. The g-ratio, on the other hand, was calculated by dividing the axon diameter (d) by the fiber diameter (D) and the coefficient of variation (CV) was obtained as standard deviation/mean x 100. These calculations were performed using Microsoft Excel.

#### Western blotting of the sciatic nerve and spinal cord proteins

Western blot was performed as described before [24]. Briefly, the sciatic nerve tissues from 3 rats/group were homogenized in RIPA buffer. The proteins measurement of the cell lysate was done using Epoch Microplate Spectrophotometer. The proteins were transferred onto PVDF membranes. The membranes were blocked with 5% milk in TBS-Tween for 1 h and incubated with the primary antibody Anti- MBP (D-18: sc-13912, molecular weight:14–22; Santa Cruz Biotechnology, Inc., Heidelberg, Germany) overnight at 4°C. The membranes were then incubated with the HRP-conjugated secondary antibody for 2 h at room temperature, washed and developed using ECL kit (RPN2109). Blots were scanned, and the band density was quantified using a densitometer (Biorad GS-800). The same protocol was applied on spinal cord tissues obtained from all experimental groups; n = 3/group, except for GBE-treated group.

#### Qualitative and quantitative analysis of spinal cord immunostained neurons

A parallel study to sciatic nerve analysis, lumbar spinal cords were also dissected and fixed by immersion overnight at 4°C, in a mixture of 4% paraformaldehyde and 0.1% glutaraldehyde in 0.2M phosphate buffer, pH 7.4. Three spinal cord tissues (L3-L6) per group (except for naive group) were processed, cut, then sectioned through multiple steps as prescribed before [28]. The protocol for histological and immunohistological staining were done following previously published work [22,29]. The primary antibodies used were Anti-NeuN (Clone A60, Mouse Monoclonal Antibody, Cat# MAB377, Millipore. Billerica, Massachusetts, USA), Anti-GFAP antibody (GA-5: sc-58766, Mouse Monoclonal, Santa Cruz Biotechnology) or Anti-GAP-43 (rabbit polyclonal IgG, ab16053, ABCAM, Cambridge, UK). The sections were observed, and the stained neurons were quantified under a light microscope. The number of Neu-N labeled neurons, GFAP immunostained astrocytes, and the intensity of GAP-43 in the ventral and dorsal grey horns of the spinal cord were determined using Cell Sens Dimension software, and analyzed as described previously [26].

#### Statistical analysis

All data were analyzed by one-way ANOVA followed by Bonferroni’s and LSD post hoc test to determine the differences in the individual baseline values using SPSS^®^ statistical program (version 22, SPSS Inc., Chicago, IL, USA). Results were considered significant when *p* < 0.05. Data were represented as the mean ± standard deviation (SD). Graphs and images were generated by Microsoft^®^ Excel and Publisher [24].

## Results and discussion

### Extraction of *Ginkgo biloba* leaves

Three kilograms of coarsely powdered *Ginkgo biloba* leaves were extracted in 80% methanol and evaporated to afford 70 g of brownish syrupy residue abbreviated GBE.

### Neuroprotective-guided fractionation of GBE

Most of the bioactive natural products were isolated using bioactivity-guided fractionation [19]. In this technique, the extract of a plant is fractionated into several fractions with different polarities based on their solubility in aqueous and organic solvents. Simultaneously, the biological activities of the fractions are tested to determine the active fraction(s). Then, the bioactive fraction is further purified into subfractions using chromatographic methods. Similarly, the purified subfractions are subjected to biological activity evaluation. This procedure allows for tracking any alteration in the bioactivity due to the purification process, which may lead to a total loss of bioactivity. It is useful, as well, to select and make changes in the purification scheme to purify the active principle(s) without significant changes in its activity.

#### Isolation, purification and identification of DGA-I-81D

Part of the obtained extract (50 g) was subjected to a vacuum liquid chromatography, where it was fractionated using increasing concentrations of MeOH in H_2_O, starting with 100% H_2_O and ending up with 100% MeOH, to afford eleven fractions with different polarities. Highly polar fractions, abbreviated DGA-I-5A, 5B, 5C, 5D and 5E, presumably contain flavonoids, were not dealt with in this study. Thin layer chromatographic analysis of the intermediate polar fractions, DGA-I-5F, 5G, 5H and 5I, suggested that they contain terpene trilactones, including ginkgolide B (Fig 2A and 2B). This analysis also showed that fractions DGA-I-5F and 5G, weighing 1.11 and 1.32 g, respectively, contain mainly ginkgolide B (R_*f*_ = 0.19) and small quantities of other ginkgolides. This assumption was further confirmed by the HPLC analysis that showed a weakly UV-active compound (λ_max_ = 219 nm), which is typical for the lactone carbonyl chromophore of the ginkgolides. Upon further purification by preparative HPLC, these two fractions afforded a pure compound, DGA-I-81D, as amorphous powder (Fig 2C). Several other methods are documented to isolate different ginkgolides using different solvent systems and chromatographic techniques [20,30].

The molecular formula of DGA-I-81D was determined as C_20_H_24_O_10_ on the basis of its molecular ion peak at *m/z* 424.1365 [M]^+^ and NMR data. On the other hand, IR spectrum showed absorption bands for hydroxyl group(s) and lactone carbonyl group. ^13^C NMR and DEPT 135° spectra (S1 and S2 Figs) showed eighteen resonances distributed as two quartets (q, CH_3_), one triplets (t, CH_2_), seven doublets (d, CH), and eight singlets (s, C). Six carbons resonated in the aliphatic region, and nine in the oxygenated carbons region (δ_C_ 69–112 ppm). The other three carbons resonated in the carbonyl groups region. The possibility of having identical carbons was confirmed by the presence of cross peaks in the HSQC (S3 Fig) spectra between δ_C_ 29.6 quartet and δ_H_ 1.13 singlet that was integrated for nine protons. This confirmed the presence of three identical methyl groups: C-18, 19 and 20. The remaining methyl group (C-16) resonated at δ_C_ 8.2, and showed cross contours with three protons resonated as a doublet at δ_H_ 1.24, in the HSQC spectra (Table 1). From COSY spectrum (S4 Fig), this methyl group, coupled to a proton, resonated at as a quartet at δ_H_ 3.03. This proton was assigned to H-14.

Additionally, ^1^H NMR spectrum (S5 Fig) showed the presence of a clear AB system. This system resonated as a pair of doublets (*J* = 7.8 Hz) at δ_H_ 4.20 and 4.59, and they were assigned as H-1 and H-2, respectively. This assignment was further confirmed by the coupling contours in COSY spectrum between these two protons. Consequently, C-1 and C-2 were unambiguously assigned at δ_C_ 75.6 and 93.5, respectively from HSQC spectra. Other COSY contours aided the unambiguous assignments of H-6 (δ_H_ 5.41), H-7 (δ_H_ 2.11 and 2.27) and H-8 (δ_H_ 1.92). On the other hand, C-2 (δ_C_ 93.5) showed three-bond cross peaks in HMBC spectra (S6 Fig), with a carbon resonated as a singlet at δ_C_ 178.5. This carbon was concluded to be C-15, which was further confirmed due to the presence of another three-bond cross peaks with H-16 (δ_H_ 1.24, d, 6.6), and two-bond cross peaks with H-14 (δ_H_ 3.03, q, 7.2). Similarly, other carbonyl groups were assigned at δ_C_ 175.4 (C-11) and δ_C_ 172.8 (C-13). The assignments of other quaternary carbons were confirmed by HMBC correlations as shown in Table 1. These spectral data constituted a solid body of evidence that unambiguously confirmed the identity of this compound as ginkgolide B. The spectral data were found to be distinguishable from those previously reported [31].

Ginkgolide B is a member of closely related compounds called diterpenoid trilactones. Their exceptional carbon skeletons are made of hexacyclic trilactones with an etheric bridge that links rings A/E to ring C, creating ring D, which makes these compounds unique to *Ginkgo biloba*.

On the other hand, other intermediate polar fractions, DGA-I-5H and 5I, were shown to be rich in all ginkgolides. Finally, the remaining two fractions, DGA-I-5J and 5K, were expected to contain the highly nonpolar constituents and were not evaluated in this study.

#### Terpene trilactones-enriched fraction

TLC analyses of the intermediate polar fractions DGA-I-5H and 5I, eluted by 70% and 80% MeOH in H_2_O, respectively, revealed that these samples contain other terpene trilactones, in addition to some ginkgolide B (Fig 2B). These fractions were pooled together with the leftover from fractions DGA-I-5F and 5G, after isolating G-B, and evaporated till dryness to give 17.3 g of terpene trilactones-enriched fraction (TEGBE). This fraction was evaluated, along with the pure G-B, for their nerve regeneration and recovery activities.

### Neurotherapeutic effects evaluation

**Fig 3:**
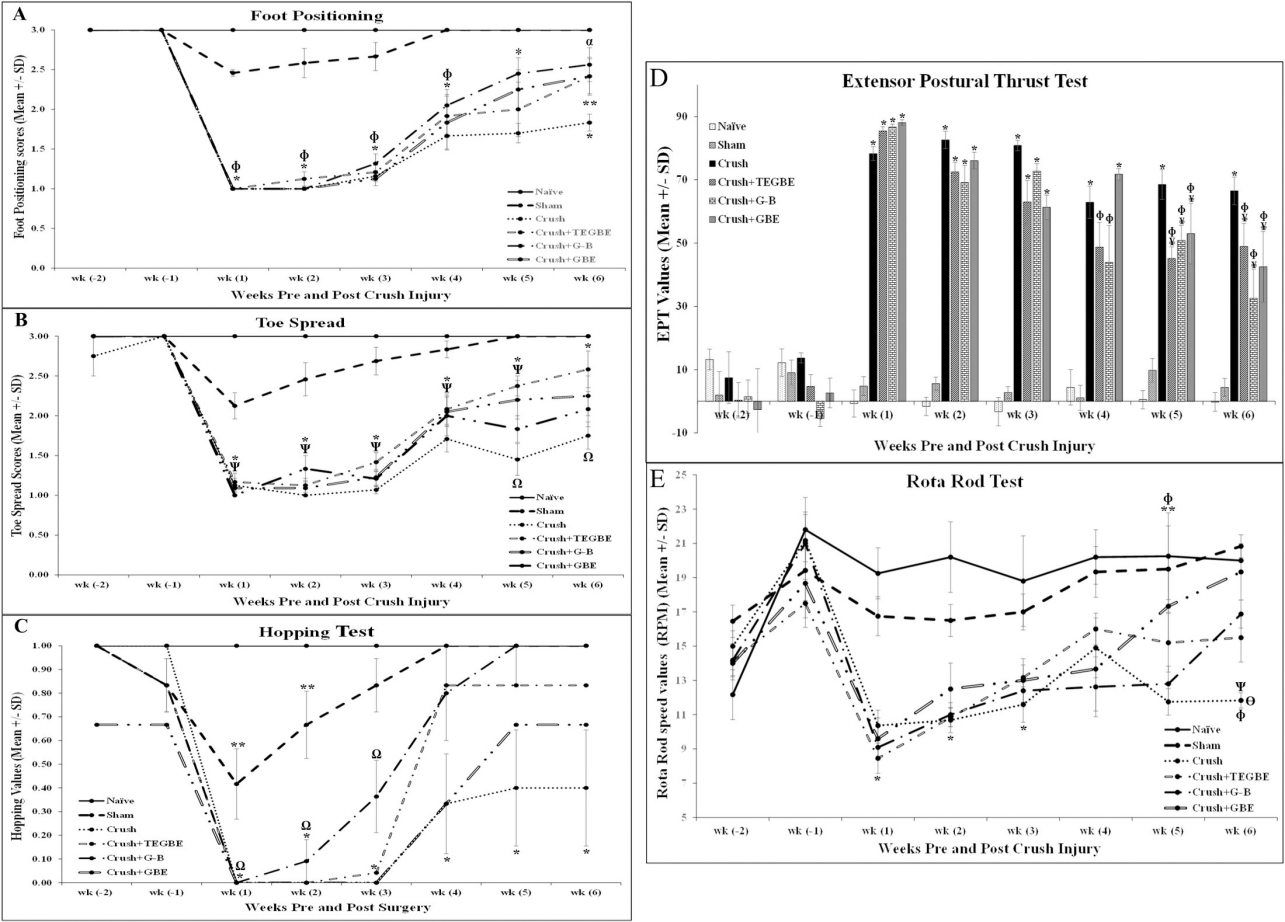
Neurobehavioral assessments. **A:** Analysis of foot position showed statistically (P< 0.05) faster and progressive recovery in TEGBE (n = 12), G-B (n = 12) and GBE-treated (n = 6) groups starting at week 4 compared to crush group. * indicates P<0.0001, crush vs. sham and naive groups. ɸ indicates P<0.002, treated groups with TEGBE, G-B and GBE vs. sham and naive groups. α indicates P<0.03, TEGBE, G-B and GBE treated groups vs. crush group. ** indicates P<0.05, TEGBE and GBE-treated groups vs. naive and sham groups. **B:** Analysis of toe spread outcome. The TEGBE, G-B and GBE-treated animals showed both clinically and statistically significant recovery in toe spread following sciatic nerve injury from week 1 till week 6 post-injury. By week 6, toe spread analysis revealed significant (p< 0.05) recovery in TEGBE-treated animals compared to crush group and is a noticeable increase in toe spread recovery of the other treated groups (G-B and GBE) toward normal values. * indicates p<0.0001, crush vs. sham and naive groups. Ω indicates P<0.003, TEGBE treated group vs. crush group. Ѱ indicates P<0.003, TEGBE, G-B and GBE treated groups vs. sham and naive groups. **C:** Differences in the mean values of the hopping test outcome among the experimental animal groups. The TEGBE and G-B-treated groups showed a significant improvement in the hopping behavior starting from week 4 until the end of the experiment compared to crush group. Further, the TEGBE and G-B-treated groups displayed an early hopping response ((as calculated by the percentage of recovered animals) at week 3 while the G-B rats started to improve by week 5. * indicates P<0.05, crush vs. sham and naive groups. ** indicates P<0.05, sham group vs. naive group. Ω indicates P<0.0001, all treated groups vs. sham and naive groups. **D:** Evaluation of functional recovery as measured by extensor postural thrust (EPT) following crush injury and TEGBE, G-B and GBE treatments. G-B-treated animals showed approximately 50% motor recovery compared to crush at week 6, indicating faster recovery. However, the G-B-treated group was not significantly recovered compared to the naive group. * indicates P<0.0001, treated groups (TEGBE, G-B, and GBE) and crush group vs. sham and naive groups. ɸ indicates P<0.05, treated groups with TEGBE, G-B and GBE vs. naive and sham. ¥ indicates P<0.05, groups treated with TEGBE, G-B and GBE vs. crush group. **E:** Motor coordination analysis is shown based on rotarod speed values following crush injury and TEGBE, G-B and GBE treatments. Rotarod performance was measured using the Rotarod test set at the acceleration mode (initial speed starts from 5 rpm/min; while maximum speed set at 45 rpm/min). The Rotarod time latency that the animal falls is indicated on the Y-axis. Pre-surgery week 2 showed less speed due to initial training and adaptation. All treated groups showed significant improvement in Rotarod performance at week 6 post-injury compared with crush group. Sham animals showed better motor coordination compared to crush and treated animals from day 1. GBE-treated animals retain their motor coordination faster than G-B and TEGBE-treated animals. However, there is no significance between treated groups by the end of week 6. * indicates p<0.05, all treated groups with TEGBE, G-B and GBE as well as crush group vs. sham and naive. ** indicates p<0.02, G-B-treated group and crush group vs. naive group. Ѱ indicates p<0.005 crush group vs. sham and naive groups. ϕ indicates p<0.05 GBE group vs. crush group. ϴ indicates p<0.05, TEGBE and G-B -treated groups vs. crush group. Values are expressed as means ± SDs.

After the induction of sciatic nerve crush injury, the hind paws of the sham-operated rats showed a normal clinical appearance throughout week 1 post-surgery, and similar to the naive animals, indicating that operative wound did not interfere with the gait and posture of the rats. In the crush and crush-treated groups, GBE, TEGBE and G-B, hind paws stayed profoundly flexed, and the animals were unable to stand throughout week 1 post-surgery. In contrast, all treated groups showed an improved clinical picture and weight-bearing characteristics during the successive weeks, and until the end of the third week. Different from GBE and TEGBE-treated groups, the G-B-treated animals recovered earlier. Additionally, neurobehavioral assessments showed in the treated groups, noticeably in G-B-treated, the foot positioning recovery started from week 4 post-injury until the end of the study as compared to the crush group (Fig 3A). Likewise, toe spread analysis revealed a faster recovery in all treated groups, particularly TEGBE-treated group which was significantly (P<0.05) improved as compared to crush animals (Fig 3B). Expectedly, G-B and GBE-treated animals regained their normal toe spread scores faster than crush animals. Treated groups showed a remarkable recovery in hopping ability as compared to crush group, P<0.05 in G-B and TEGBE-treated animals in week 4 post injury (Fig 3C). In the extensor postural thrust test (EPT) (Fig 3D), G-B-treated animals displayed faster recovery than TEGBE and GBE-treated and demonstrated significant (*p*< 0.05) decrease in the functional motor deficits by the end of week 5 as compared to crush animals. Rotarod test analysis revealed that all treated groups recovered faster as compared to crush group, starting from week 4, and profoundly in GBE-treated group at week 5 (P<0.05) followed by G-B and TEGBE at week 6 (Fig 3E). In contrast, crush animals showed severe motor coordination disabilities as compared to sham animals in day 1 post-injury and until week 6 (*p<* 0.0001), indicating a profound injury to motor neurons. Similar effects by GBE were previously reported [32] on a transient sciatic nerve injury, but not in a sciatic crush injury model.

**Fig 4:**
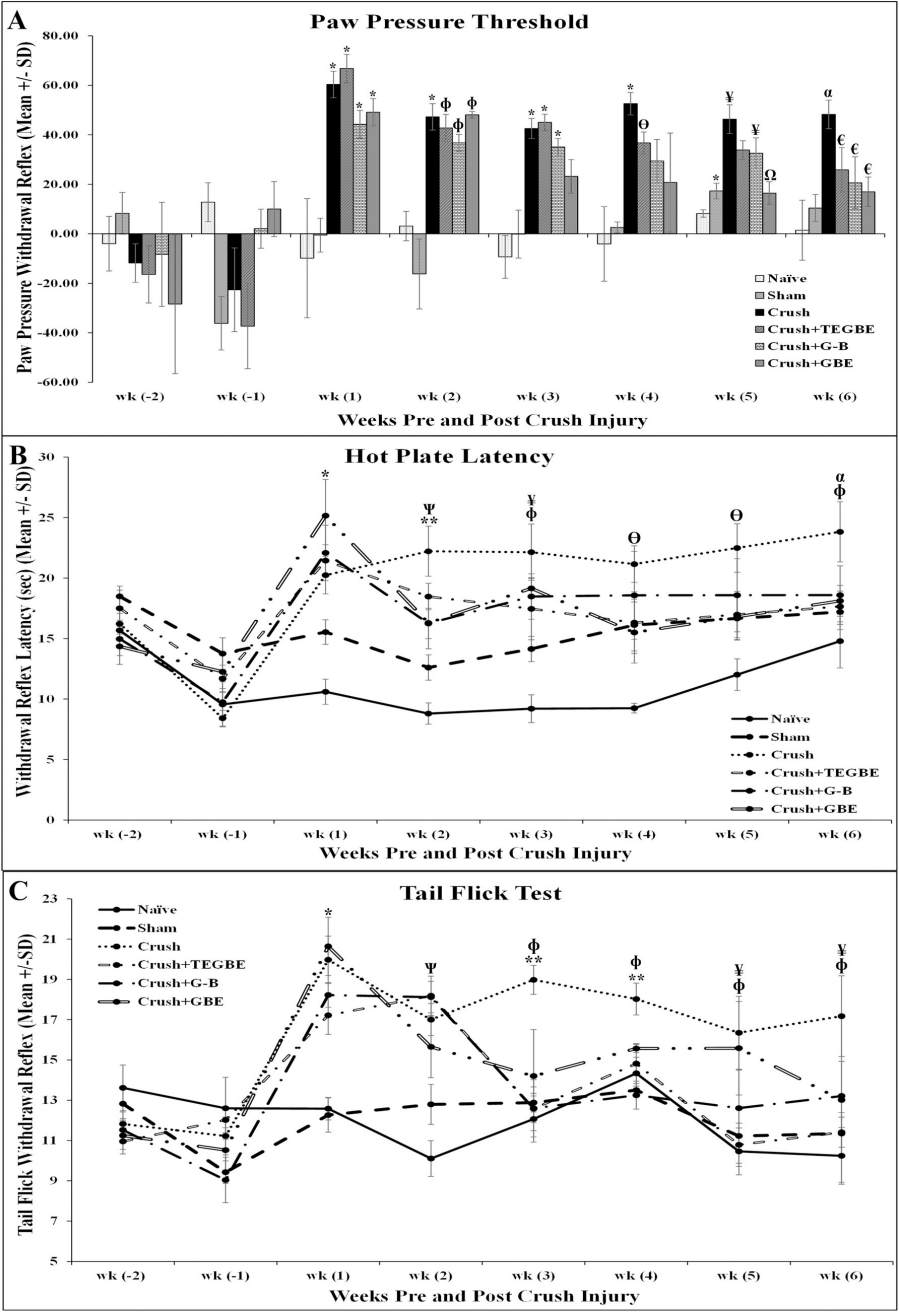
Sensory tests. **A:** Nociceptive mechanical thresholds expressed in grams were measured with an analgesic meter, 2 weeks before nerve crush injury till week 6 post-surgery. As time progressed, all treated rats showed trending recovery toward value = 0, indicating that mechanical hyperalgesia as measured by paw withdrawal reflex to nociceptive mechanical pressure was affected by all treatments compared to crush animals (n = 12). GBE (n = 6) was most effective treatment, by the end of week 6 with a significant decrease in PPT latency. PPT latencies were significantly increased in crush animals compared to sham (n = 12) by the end of week 6. * indicates p<0.0001, crush group and treated groups (TEGBE (n = 12), G-B (n = 12) and GBE) vs. naive (n = 6) and sham groups. ɸ indicates p<0.01, all treated groups with TEGBE, G-B and GBE vs. naive and sham groups. ϴ indicates p<0.03, TEGBE treated group vs. naive group. ¥ indicates p<0.05, TEGBE and G-B treated groups vs. naive and sham groups. Ω indicates p<0.01, GBE treated group vs. crush group. α indicates p<0.05, crush group vs. naive and sham groups. € indicates p<0.05, all treated groups (TEGBE, G-B and GBE) vs. crush group. **B:** Withdrawal reflex latency performed 2 weeks pre-surgery until week 6 post-surgery. Withdrawal reflex is defined as the time elapsed from the onset of hotplate contact to the withdrawal of the hind paw and measured in seconds. The affected limbs were tested twice with a one-hour interval between consecutive tests to prevent any sanitization or injury. The average readings were reported to obtain the final result. The cut-off time for heat stimulation was set at 30 s to avoid skin damage to the foot. Nerve crush injury produced a severe nociception deficit in the crush and crush-treated groups at week two following nerve injury. The TEGBE, G-B and GBE-treated animals showed a significant thermal nociceptive recovery by week 2 post-injury. * indicates p<0.05, all treated groups (TEGBE, G-B and GBE) and crush group vs. naive and sham groups. ** indicates p<0.05, TEGBE-treated group and crush group vs. naive and sham groups. Ѱ indicates p<0.05, G-B and GBE-treated groups vs. crush group. ɸ indicates p<0.05, crush group vs. naive and sham groups. ¥ indicates p<0.05, all treated groups (TEGBE, G-B and GBE) vs. naive group. ϴ indicates p<0.05, sham, crush and G-B -treated groups vs. naive group. α indicates p<0.05, TEGBE-treated group vs. crush group. Results are presented as mean ± SD. **C:** Time course of tail flick withdrawal latency in the different experimental groups. The spinally mediated nociceptive thresholds were determined by using an analgesia meter apparatus weekly from week 2 pre-surgery week till the end of week 6 post-surgery. The time taken for the tail to move was recorded in seconds as tail flick withdrawal reflex. The cutoff time was set on 25 sec to avoid any tissue damage. Two readings were taken and the all averages were obtained for the final values. Crush animals displayed significantly higher values for tail flick withdrawal latencies compared to naive and sham groups throughout the experiment. Whereas, The TEGBE, G-B and GBE-treated groups showed significant tail flick withdrawal latency recovery compared crush group by the end of week 3 post-injury. * indicates p<0.005, all treated groups (TEGBE, G-B and GBE) and crush group vs. naive and sham groups. Ѱ indicates p<0.05, crush group and TEGBE treated group vs. naive and sham groups. ɸ indicates P<0.05, crush group vs. naive and sham groups. ** indicates p<0.05, all treated groups (TEGBE, G-B and GBE) vs. crush group. ¥ indicates p<0.05, TEGBE treated group vs. crush group. Values are expressed as means ± SDs.

Sensory tests, on the other hand, including paw pressure test (PPT, mechanical hyperalgesia), tail flick test and hot plate test (thermal hyperalgesia) revealed that treated animals exhibited improvement in their thermal, mechanical and central latencies (Fig 3). Previous investigations [21,33] indicated that GBE is an effective remedy, as compared to the control group, based on sensory analysis of neuropathic parameters. However, these findings have been documented on GBE only and not on the trilactones-enriched extract (TEGBE) or pure G-B. In the present study, PPT findings revealed that GBE-treated animals recovered faster than TEGBE and G-B, however, they showed no significance (*P*>0.05) by the end of week 6 (Fig 4A). On the other hand, the mean baseline of thermal hyperalgesia (withdrawal reflex latency) of crush animals was significantly greater as compared to sham animals (*p*<0.05) (Fig 4B). However, treatment with GBE, TEGBE or G-B prevented the thermal hyperalgesia. There was no significant (*P*>0.05) difference in paw withdrawal latency in hot plate test between all treated animals throughout the second week post-surgery. The mean tail flick latency of sham (12.26±0.85 sec) and naive (12.58±0.55 sec) rats showed no significant difference on week 1 post-surgery until the day of sacrifice (*p*>0.05) (Fig 4C). GBE, TEGBE and G-B treatments significantly (*P*<0.05) prevented the spinally mediated thermal hyperalgesia as compared to the crush rats by week 3 post-surgery.

However, by end of week 6, tail flick withdrawal reflex was totally regained in all treated groups as compared to sham and naive groups (P>0.05). Functional and sensory deficits in crush group in this study were consistent with our previous data, indicating the successful reproduction of the sciatic crush injury model [22]. Further, enhancements of functional and sensory recovery in the GBE-treated sciatic crush injury animals were in agreement with previously reported work on GBE treatment in facial crush injury model [34]. Consistent with the current findings, a published study [35] reported that GBE was effective in protecting ganglion cells in optic nerves following crush injury.

Despite that all treatments (GBE, TEGBE and G-B) showed positive effects on neurobehavioral tests when compared to the control crush group, G-B and TEGBE modulated the motor and sensory tests more effectively than did GBE. This indicates that terpene trilactones, i.e., ginkgolides, might have a more critical impact on the recovery of the sciatic nerve crush injury than the flavonoids in GBE do. The current study also examined the role of G-B in sciatic nerve recovery following crush injury, as there were no previous reports on either TEGBE or G-B. Thus, the current research intended to study the neuroprotective implications of a pure ginkgolide, i.e., G-B, and other ginkgolides, in one treatment (TEGBE), following nerve crush injury.

### Light and transmission electron microscopic (TEM) examinations

**Fig 5:**
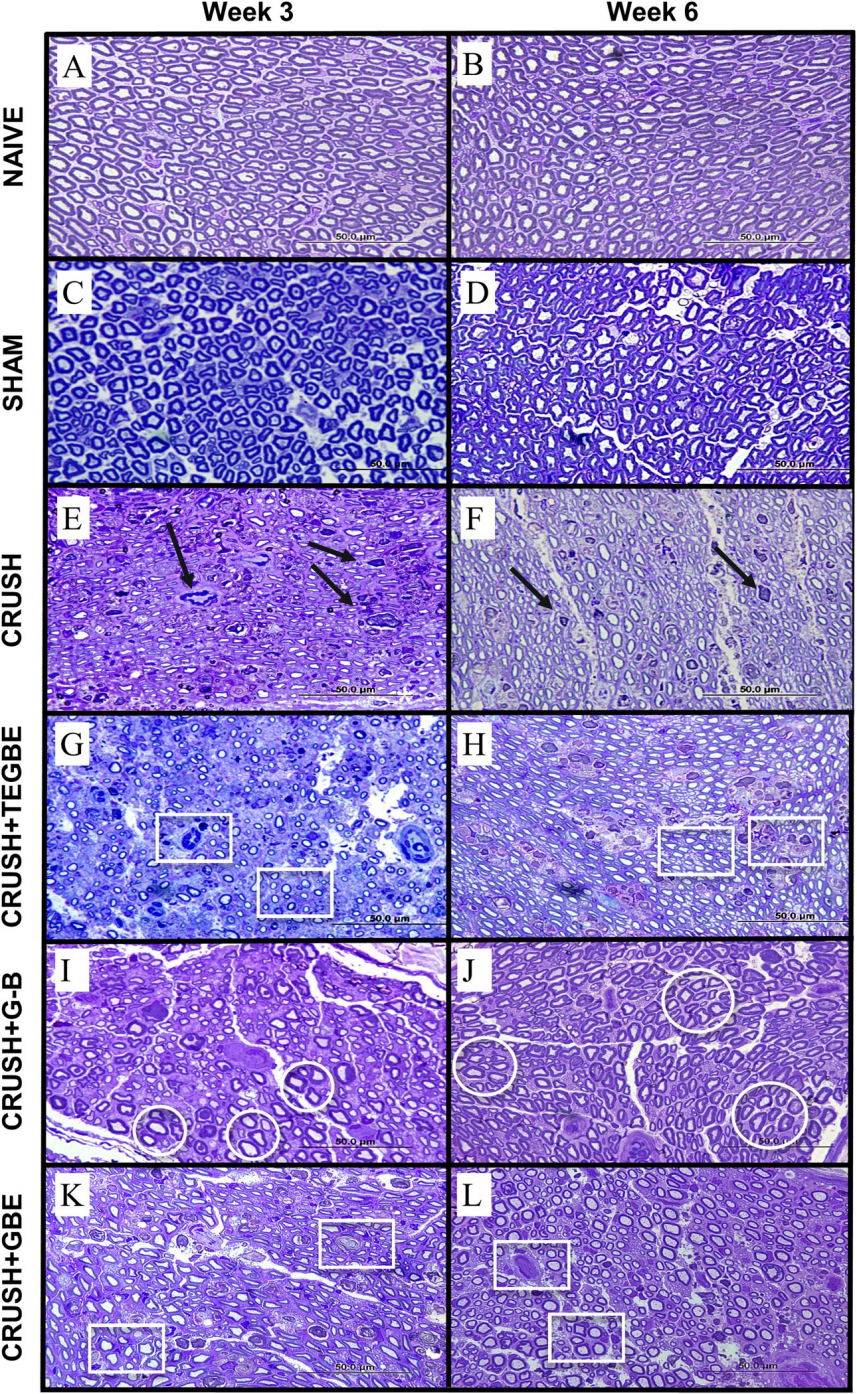
Histological examinations. Toluidine blue stained photomicrographs of semi-thin transverse sections of sciatic nerves obtained from animals at week 3 and week 6 after crush injury, respectively. Specimens acquired from animals in the naive (n = 6) (**A, B**), sham (n = 12) (**C, D**), crush (n = 12) (**E, F**), TEGBE-treated (n = 12) (**G, H**), G-B-treated (n = 12) (**I, J**) and GBE-treated (n = 6) (**K, L**) groups. Naive (**A, B**) and sham (**C, D**) groups display normal nerve fibers and axonal morphology appearance and axons are surrounded by myelinated fibers compared to crush (**E, F**). The crush animals show axonal disintegration, phagocytosis and a lot of myelin and fiber debris among unmyelinated axons with the presence of smaller mini-fascicles nerve fibers with less myelin and macrophages filled with degraded myelin (arrows). At week 3, the TEGBE-treated group (**G**) exhibits a remarkable recovery of most of the axons and acceleration of myelination process. The sciatic nerve sections of G-B-treated rats show much more improvements in re-myelination of axons compared to the two other treated groups (**G, K**) (circles). In GBE-treated rats (**K**) smaller and thinner axons exist in addition to the debris of phagocytotic processes (rectangles). At week 6 post-injury, the all the treated animals (**H, J and L**) display approximately normal myelinated axons with a noticeable increase in myelin layers compared with those from the crush (**F**). Further, G-B-treated (**J**) group shows less number and density in regenerated myelin axons compare to H and L (circles) (1000X Magnification).

**Fig 6:**
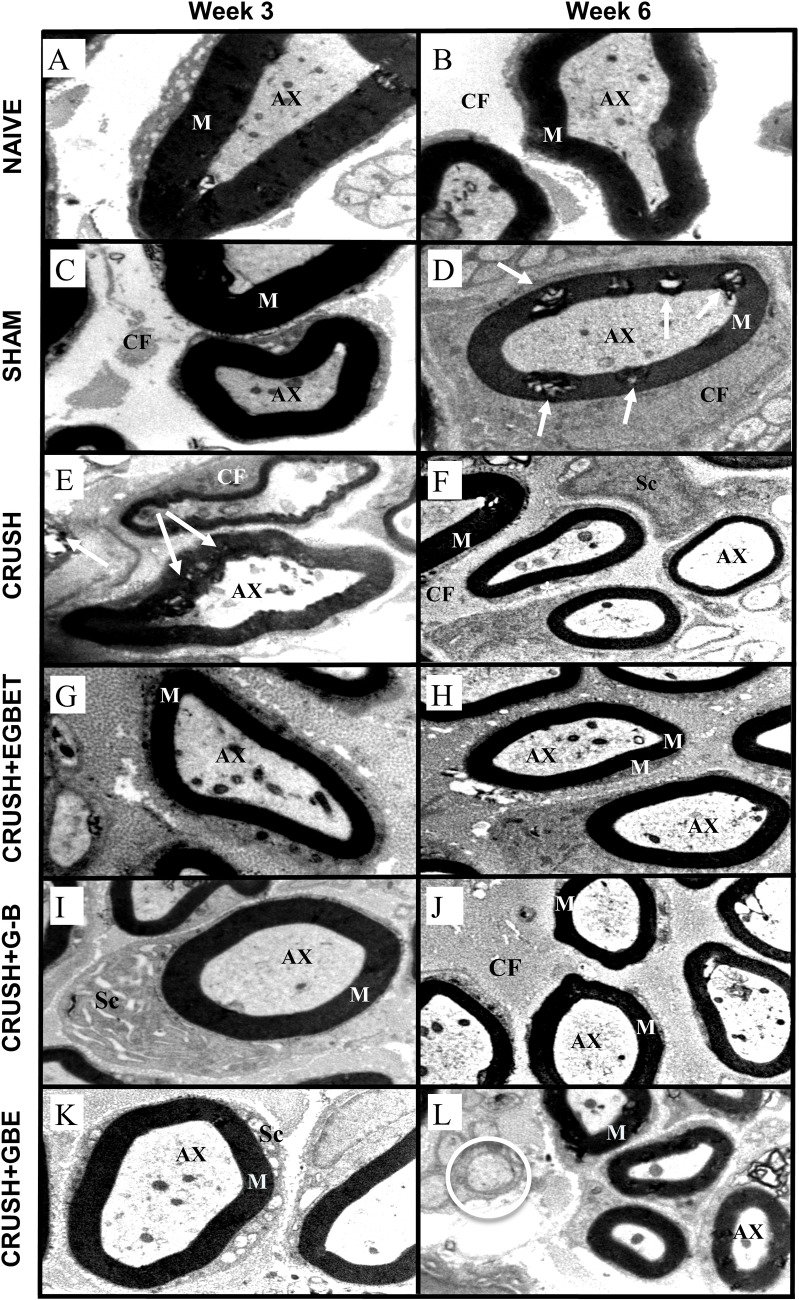
Electron microscopy. Sciatic nerve electron micrographs from the different experimental groups at week 3 and week 6 post-injury (15000x). Naive (n = 6) (**A and B**) and sham (n = 12) (**C and D**) groups clearly display normal and healthy myelin sheaths (M) and axonal fibers (AX) along with stable Schwann cells (Sc) at week 3 and week 6, respectively. The crush animals (n = 12) (**E**) show the unhealthy appearance of myelin sheaths (arrows) and irregular shape of nerve fibers abnormally presented vacuoles in addition to disorganized collagen fibers at week 3 post-injury. Likewise, at week 6 post-injury, the crush animals (**F**) still exhibit abnormal, collapsed and fibrillated Schmidt-Lantermann clefts (small arrows) with irregular and intermingled myelin sheaths and axons (AX). In contrast, at week 3 post sciatic nerve injury, the TEGBE (n = 12) (**G**) G-B (n = 12) (**I**) and GBE-treated (n = 6) animals (**K**) show healthy myelin sheaths with normal appearance of nerve fibers and typical arrangement of collagen fibers (CF) along with normally looking Schwann cells (Sc). The GBE-treated animals (**K**) display healthy and normal myelin sheaths and axonal fibers as well as the existence of smaller and thinner myelinated nerve fibers. Likewise, at week 6 post-injury, all the treated groups (**H, J and L**) show the normal and healthy appearance of myelin sheaths along with distinctively organized distribution in the extracellular matrix (EM) with clear Schmidt-Lantermann clefts and healthy and regularly arranged collagen fibers (CF).

Histological examinations of the toluidine blue-stained sciatic nerve specimens of sham and naive groups revealed a normal anatomical appearance of sciatic nerves, with regular distribution of small and large diameter nerve fibers, as well as normal proportion between myelin sheath thickness and fiber diameter at both 3 weeks and 6 weeks post-surgery (Fig 5A–5D). These observations of naive and sham groups were confirmed by electron microscopy, which showed healthy myelin sheaths thickness and typical arrangement of collagen fibers at both time intervals (Fig 6A1–6A4 and 6B1–6B4).

Additionally, the crush group sections displayed Wallerian degeneration features (Fig 5E) at week 3 post-surgery. Collagen fibers in endoneurium showed large separations and discontinuations within the nerve bundles. Also, unmyelinated and irregular axons were abundant as well as the disintegration of axonal cytoskeleton elements in week 3. By the end of week 6 post-surgery (Fig 5E), crush sections showed predominantly newly-regenerated thin myelinated nerve fibers with widespread myelin sheath degeneration. The morphological and ultrastructural analysis of the crush group illustrated a diminished nerve regeneration process, a decrease in macrophage recruitment, reduced activation of phagocytic Schwann cells, and a decline in Wallerian degeneration, even after 6 weeks post-injury. These findings were similar to those previously described [29].

Crush sections at week 3 post-surgery, in electron microscopy, displayed irregularly shaped and highly condensed abnormal myelin sheaths and axonal degradations (Figs 6 and S7). At week 6 after crush injury, however, crush sections exhibited newly regenerated nerve fibers, organized in small fascicles, which were characterized by smaller size and thinner myelin sheaths as compared to sham sections. There was some existence of degenerative fibers among newly regenerated ones, indicating that regeneration was still in progress at week 6 (Fig 6E and 6F). These findings are consistent with other studies [25] supporting the current findings that even after 6 weeks post-injury the process of maturation of regenerated nerve fibers is still incomplete and very slow, when compared to groups treated with GBE, TEGBE and G-B.

In contrast, sciatic nerve specimens of animals treated with G-B, TEGBE and GBE showed a remarkable axonal and myelin regeneration with more newly regenerated nerve fibers as compared to crush sections at week 3 post-injury (Fig 5G, 5I and 5K). The ultrastructural changes of the sciatic nerve for animals treated with G-B improved largely as compared to TEGBE and GBE treated animals. At week 6, the tissue specimens of the treated animals with G-B, TEGBE and GBE (Fig 5H, 5J and 5L) showed almost normal morphological appearance of sciatic nerves as compared to sham and naive groups (Fig 5B and 5D). Further, the number and density of regenerated axons in G-B-treated rats was distinctly lower than other treated groups (TEGBE and GBE) (Fig 5J, 5H and 5L). Evidently, TEM sections of sciatic nerves from animals treated with G-B, TEGBE and GBE showed that nerve fibers were recovered following sciatic crush lesion at week 3, and improved as time progressed (Figs 6G, 6I and 6K and S7). At week 3, thin and regular myelin sheaths were present predominantly with the absence of disintegrated myelin figures as in crush group. Moreover, the extracellular matrix of TEM sections obtained from treated groups displayed normal appearance of collagen fibers as compared to crush group (Figs 6E and S7). At week 6 following crush injury, treatments with G-B, TEGBE and GBE successfully recovered the Schmidt-Lantermann clefts associated with a typical and healthy-looking myelin sheath (Figs 6H, 6J and 6L and S7). Findings from light and electron microscopic examinations showed that morphological alterations by G-B, TEGBE and GBE had an important role in the functional recovery of the sciatic nerve. The current data support other recent *in vitro* and *in vivo* studies that showed GBE has a critical modulation on axonal regeneration and Schwann cell activation [36,37]. Furthermore, the positive effects of GBE on sciatic nerve regeneration and neovascularization in current findings were in agreement with many reports [17,18].

### Morphometric analysis

**Fig 7:**
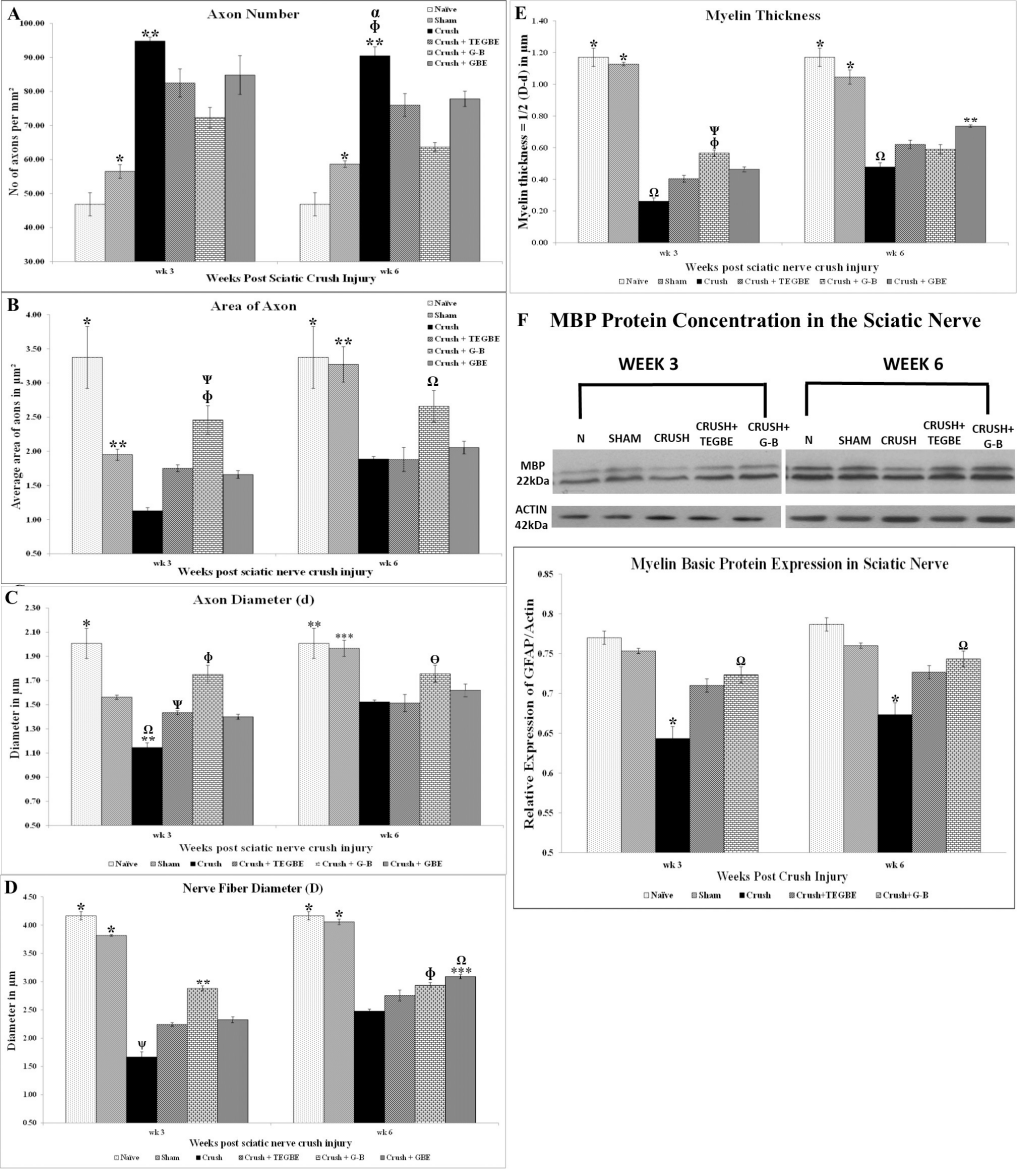
Morphometric analysis. **A:** Mean total numbers of myelinated axons (Number of axons/field at 1000x magnification) counted from 4 different random samples from each group used in the morphometric study. The mean total number of myelinated axons was calculated for each experimental group at week 3 and 6 following sciatic nerve injury using a 1 mm^2^ square counting frame. Note the all the treatments significantly decreased the number of axons/field following nerve crush injury at Week 3 and week 6. * indicates p<0.0001, sham (n = 12) vs. crush group (n = 12), TEGBE (n = 12) and GBE (n = 6) treated groups. ** indicates p<0.0001 crush vs. naive (n = 6), sham and G-B treated group (n = 12). ɸ indicates p<0.005, crush vs. TEGBE treated group. α indicates p<0.02, crush vs. GBE treated group. Values are expressed as means ±SD. **B:** Mean cross-sectional areas of myelinated axons obtained from 4 different and randomly selected samples from each group (3 animals/group). The averaged cross-sectional areas of myelinated axons measured in square micrometers (μm2) were calculated for each experimental group at week 3 and 6 following sciatic nerve injuries. Compared with crush group, G-B-treated animals show significantly higher in the average areas of axons per unit square among other treated groups at week 3 and 6, p<0.0001 and p<0.02 respectively. * indicates p<0.0001, naive vs. crush, TEGBE treated groups. ** indicates p<0.005, sham vs. crush group. ɸ indicates p<0.0001, G-B treated group vs. crush. Ω indicates p<0.02, G-B-treated group vs. crush and TEGBE-treated groups. Ѱ indicates p<0.005 G-B vs. GBE treated animals. The values are expressed in mean ± SDs. **C, D, E:** Stereological estimation of the myelinated axon diameter **(C)**, myelinated nerve fiber diameter **(D)**, and myelin thickness **(E)** obtained from 4 different random samples from each of 3 animals per group. The mean values of the axon (d) and nerve fiber (D) diameters were calculated using an image analysis software (Image Pro-Plus 6.0) for each experimental group at weeks 3 and 6 following sciatic nerve injuries using a square counting frame. **C:** The crush and treated groups display significantly lower in myelinated axon diameter compared to all groups compared to naive and sham animals at week 3 and week 6 post-injury, whereas the G-B-treated group display significantly high in the mean of myelinated axon diameter among other treated groups at both time points. * indicated p<0.0001, naive vs. sham, crush, TEGBE and GBE-treated groups. ** indicates p<0.0001, crush vs. naive, sham, and G-B. Ω indicates p<0.005, crush vs. TEGBE and GBE-treated groups. ɸ indicates p<0.0001, G-B-treated group vs. crush and GBE. Ѱ indicates p<0.002, TEGBE treated group vs. crush and G-B-treated groups. *** indicates p<0.0001, sham and naive vs. crush and TEGBE-treated groups. ϴ indicates P<0.03, G-B-treated group vs. crush and TEGBE-treated groups. **D:** The crush group displays a significant decrease in nerve fiber diameter at both time points assessed following nerve injury compared with sham and naive groups. However, G-B treated group exhibits a significantly increase in nerve fiber diameter among other groups compared to crush group at week 3 post injury. Whereas, TEGBE-treated group shows a significant increase in the nerve fiber diameter only at week 6 compared to other groups. * indicates p<0.0001, naive and sham compared to all other groups at week 3 and week 6. ** indicates p<0.0001, G-B vs. all other groups (higher than crush group, TEGBE and GBE-treated groups and lower than naive and sham groups). Ψ indicates p<0.0001, crush vs. all other groups. ɸ indicates p<0.0001, G-B treated group vs. naive, sham and crush groups. *** indicates p<0.0001, GBE vs. naive, sham and crush groups. Ω indicates p<0.02, GBE vs. TEGBE. **E:** The average values of myelin thickness were obtained from (D) and (d) values and calculated by the following formula: ½(D-d). The calculated myelin thickness for the crush and the treated groups (TEGBE, G-B and GBE) at week 3 and week 6 following sciatic nerve injury shows a significant decrease in a myelin thickness throughout the experiment compared to naive and sham groups. However, the myelin thickness in all treated groups increased significantly compared with crush group at week 3 and week 6 post-injury. * indicates p<0.0001, naive and sham vs. crush and treated groups with TEGBE, G-B and GBE. ɸ indicates p<0.0001, G-B-treated group vs. crush group. Ѱ indicates p<0.005, G-B-treated group vs. TEGBE treated group. ** indicates p<0.0001, GBE treated group vs. crush. Ω indicates P<0.02, crush group vs. all groups. Values are expressed as means ±SDs. **F:** Western blotting graph and data analysis of myelin basic protein (MBP) expression in the sciatic nerves obtained from naive, sham, crush, crush + G-B and crush + TEGBE and normalized with densities of actin band (Molecular weight-42). GBE treated group was not applied to this test due to a shortage of animal supply. In each group, sciatic nerve from three rats was analyzed. The antibody used to detect the MBP (D-18): sc-13912, Santa Cruz Biotechnology, Molecular weight-14-22) recognizes three distinct bands. Note that the MBP was significantly (*p<0.0001) decreased in crush group compared to all groups at weeks 3 and 6 following sciatic nerve injury. Also, MBP was significantly (Ω p<0.05) increased in G-B treated group compared to TEGBE treated group at both time points. Values are expressed as means ± SDs.

Quantitative stereological analysis of sciatic nerve after 3 and 6 weeks post-surgery are presented in Fig 7A–7E.

The results from morphological parameters (axon numbers, areas, and diameters, nerve fiber diameter and myelin thickness) per unit area of the sciatic nerve cross sections obtained from all groups, supported the qualitative data from microscopic examinations. Mean axon numbers per unit nerve area (Fig 7A) were significantly (*P*<0.0001) lower in G-B-treated group as compared to crush group at week 3 post-injury, but no difference was shown as compared to other treated groups. However, at week 6, G-B-treated animals showed lower mean axon numbers than other treated groups, but equivalent to sham group. Also, the mean axon area per unit nerve area was significantly (P<0.0001) higher in G-B-treated group than crush group, but no significant difference (P>0.05) as compared to sham, TEGBE and GBE-treated groups at week 3 post-injury (Fig 7B). At week 6 post-surgery, the average area of axons per unit nerve area in sections obtained from G-B-treated rats was equivalent to sham and naive (*p*>0.05) and significantly (*p*<0.05) was higher than crush group, and unlike TEGBE, and GBE-treated groups that showed no significant difference in their myelinated axon area (*p*>0.05) as compared to crush group.

The axon diameter sizes in all treated groups were equivalent (*P*>0.05) to sham, but significantly (*P*<0.01) higher than those in crush group at 3 weeks post-surgery (Fig 7C). At 6 weeks, the axon diameter size in G-B-treated group was significantly higher than that in the crush group (*P*<0.05) as compared to other treated groups, which were not more significant than crush group (P>0.05). Further, G-B-treated animals were not significantly different than the sham group (*P*>0.05).

Similar to axon diameter, nerve fiber diameter was significantly (*P<*0.0001) high in naive and sham groups as compared to the crush group at both time points (Fig 7D). All treated groups also displayed significantly (*P*<0.0001) higher nerve fiber diameter per unit area as compared to the crush group, however, it was significantly (*P*<0.0001) lower as compared to the sham and naive groups at week 3 post-surgery. At week 6 post-injury, nerve fiber diameters obtained from G-B and GBE-treated groups were significantly (*P*<0.01) higher than those in the crush group, however, TEGBE-treated rats showed no significant (*P*>0.05) difference when compared to the crush group at week 6 (Fig 7D).

The calculated myelin thickness for the crush and all treated groups at both time points following sciatic nerve injury showed a significant (*p*<0.0001) decrease as compared to the naive and sham groups (Fig 7E). However, myelin thickness obtained from G-B and GBE-treated groups showed a significant increase (*p*<0.0001) as compared to the crush group at both time points. Seemingly, TEGBE-treated group displayed no significance as compared to crush group at both time points.

Only one study reported the positive effects of GBE on nerve regeneration and functional recovery in an animal model of crush sciatic injury [38]. However, this study lacks the histological and immunohistochemical analyses of the spinal cord following injury. Also, no thorough investigation on neurotherapeutic effects of GBE isolated constituents, such as G-B, was reported in that study.

**Fig 8:**
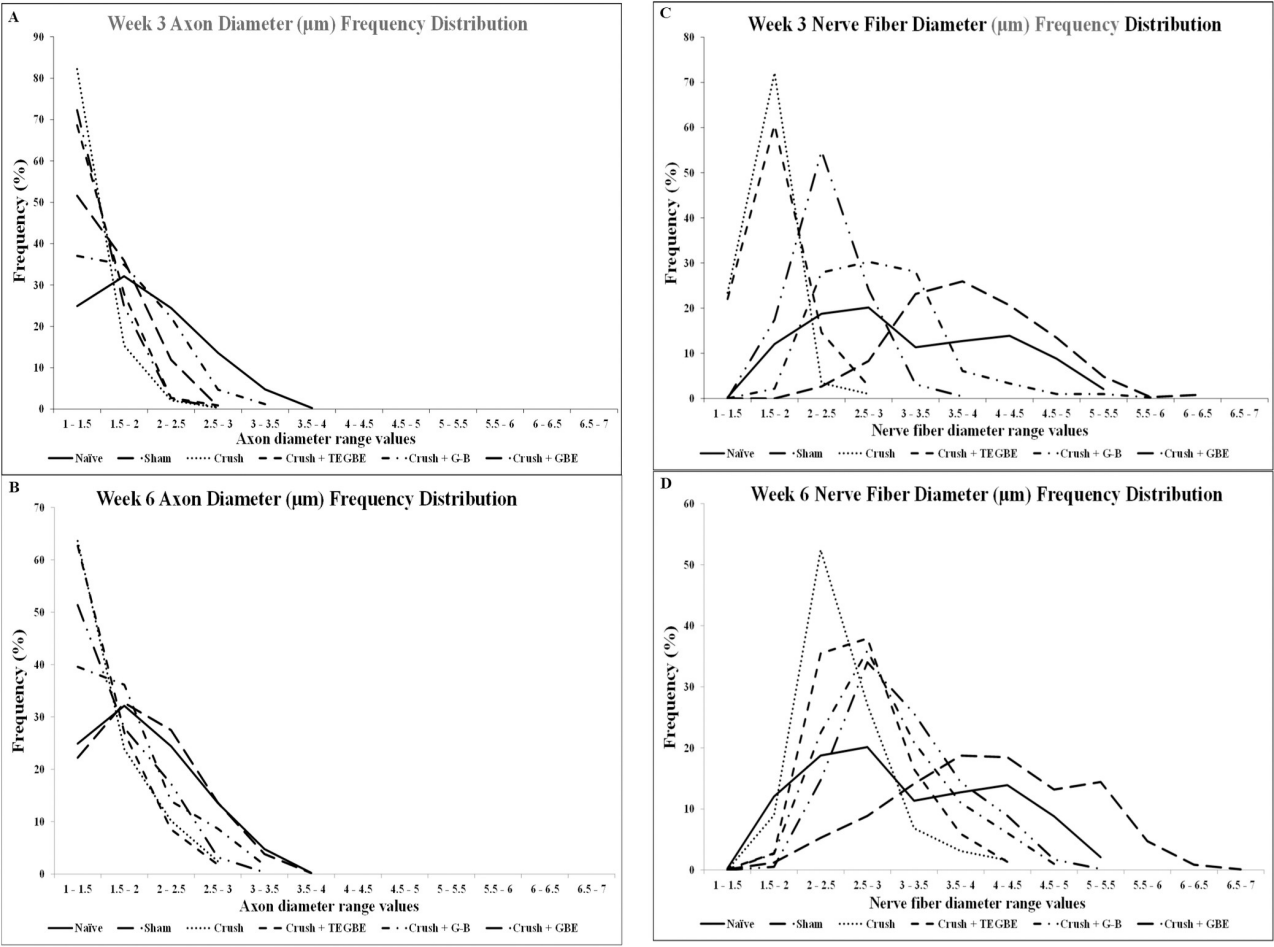
Axons and nerve fibers distribution. It is showing the frequency of the distributions of the diameters of myelinated axons and nerve fibers at week 3 **(A, C)** and week 6 **(B, D)** of sciatic crush nerve injury, respectively, which further confirm the above morphometric findings. The percentage frequency of the myelinated axons and nerve fibers (y-axis) were plotted against the diameter sizes (x-axis) ranging from 1 μm 10.5 μm from the experimental. Naive (n = 6) and sham (n = 12) groups show normal frequency distribution of myelinated axon **(A, B)** and nerve fiber **(C, D**) diameters unlike the crush group (n = 12) at 3 and 6 weeks post-injury which show a remarkable shift in the distribution of the axon and never fiber diameter sized to the left toward low range values compared to naive and sham animals. In general, all the treated groups showed a remarkable right shift in both axon and nerve fiber frequency distributions toward normalization. Interestingly, the G-B-treated group (n = 12) shows faster influence on the frequency distribution shift towards normal values compared to other treated groups at both time points.

As such, further confirmation of the morphometric findings was obtained from the axon and nerve fiber diameter distribution at weeks 3 and 6 post injury (Fig 8A–8D).

The crush group displayed a remarkable shift to the left in the distribution of both axon and nerve fiber diameters as compared to the sham animals at week 3 and week 6. Although all the treated groups showed an improvement pattern of the axon and nerve fiber diameters distributions, the G-B-treated group displayed more normalization of axon and nerve fiber diameters distributions as compared to TEGBE and GBE-treated groups at week 3 and week 6. So, according to the qualitative and quantitative analysis, crushed sciatic nerves in animals treated with G-B were recovered in a quick manner as compared to other treated groups. These findings strongly support that G-B effects on sciatic nerve regeneration might be through the involvement PAF mechanism. Additionally, this study showed that the TEM ultrastructural findings were coherent with those obtained from the stereological analysis on the sciatic nerve following the administration of GBE, TEGBE, and G-B treatments.

As supportive data to the stereological analysis, western blotting of myelin basic protein (MBP) in sciatic nerves obtained from naive, sham, crush, G-B and TEGBE-treated groups showed that treatment with G-B and TEGBE (Fig 7F) either protected or increased the MBP as compared to the crush group. A significant (*P*<0.0001) decrease in the MBP content in the crush as compared to all other groups was shown. In contrast, sciatic nerves from G-B-treated group exhibited significantly (*P*<0.05) high MBP content as compared to TEGBE-treated group. Western blotting of MBP was also performed to confirm the morphological analysis in sciatic nerve injury treated with G-B as compared to the crush, sham, and naive groups.

### Nissl staining

**Fig 9:**
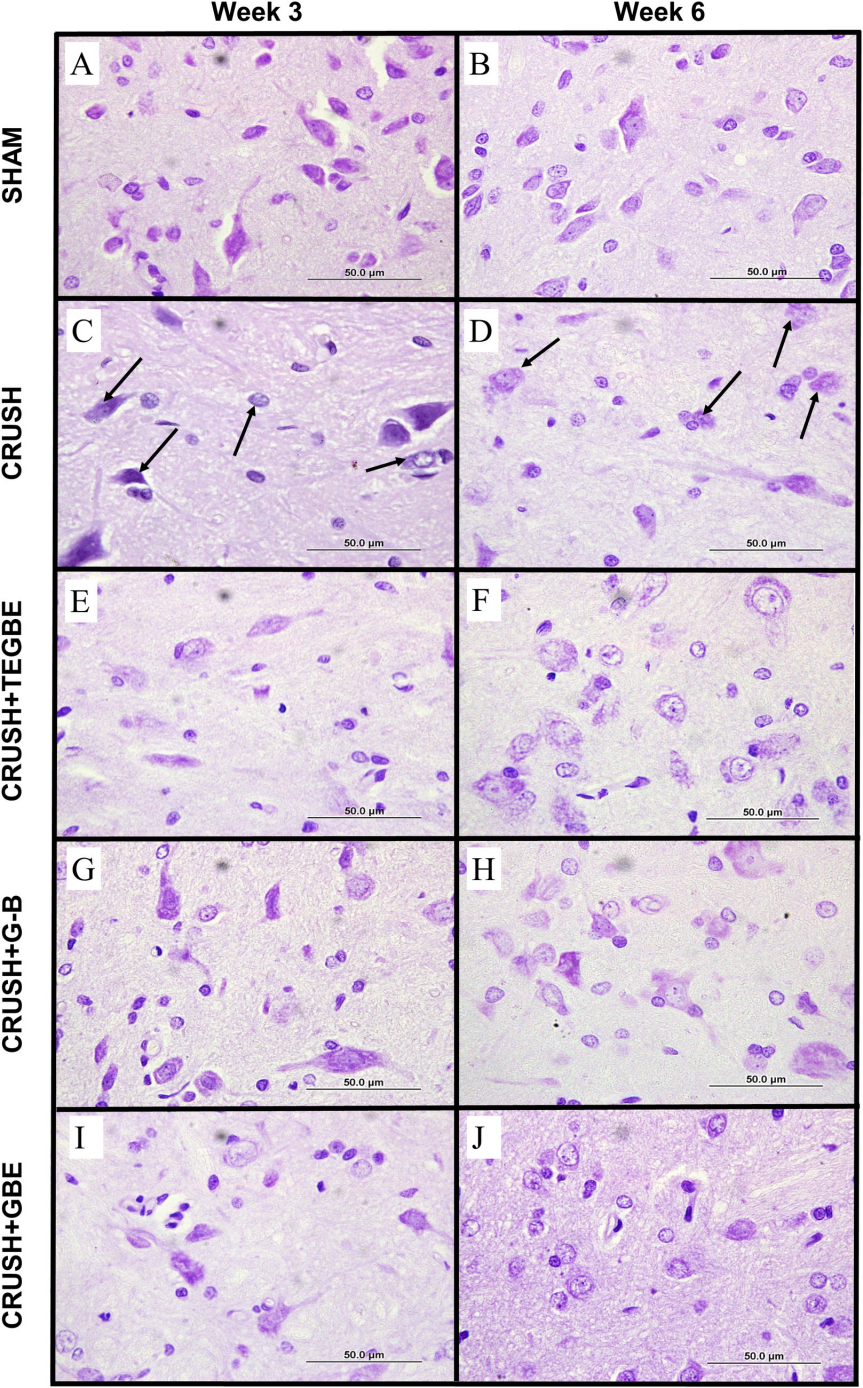
Nissl staining. Representative photomicrographs (100x objectives) of the ipsilateral dorsal horn of the L3-L6 spinal cord segments stained with cresyl violet from all groups at weeks 3 and 6 following sciatic nerve injury. Sham group (n = 12) (**A, B**) shows normal sensory neurons with stained Nissl bodies. Crush group (n = 12) **(C, D)** displays less and chromatolysed neurons compared to sham group (arrows). TEGBE (n = 12) **(E**, **F)**, G-B (n = 12) **(G, H)** and GBE-treated (n = 6) **(I, J)** groups display remarkably more protected sensory neurons with normal morphology and Nissel Bodies staining compared to crush group at week 3 and week 6 post-injury.

A qualitative analysis of cresyl-violet-stained spinal cord sections clearly showed that Nissl substances in the crushed sections were absent in most of the dorsal (sensory) (Fig 9C and 9D) and ventral (motor) neurons along with the chromatolysed neurons.

Moreover, the crush cross-sections displayed abundant irregular neurons and eccentric nucleus of motor neurons in the ventral horn at week 3 following injury. Further, sections obtained at week 6 showed remarkable Nissl-devoid neurons as compared to sham group. In contrast, sham group showed normal distribution of Nissl substance in all neurons with clear nuclei in both dorsal and ventral horns at week 3 and week 6 post-injury.

The spinal cord sections obtained from treated groups with G-B, TEGBE and GBE at week 3 following sciatic injury displayed more recovered Nissl stained neurons than the crush group, and more abundant trapezoidal shape of motor neurons in ventral horn. However, dorsal neurons in G-B-treated group (Fig 9) exhibited a noticeably higher number than other treated groups (Fig 9E and 9I) at week 3 post-injury. At week 6 post-injury, sections obtained from the treated groups displayed much more Nissl stained neurons as compared to crush spinal cord sections (Fig 9F, 9H and 9J). These current findings were in agreement with previous investigations using Nissl staining analysis on G-B [39] and GBE [40] treated models of spinal cord injury.

### Immunohistochemical analysis

**Fig 10:**
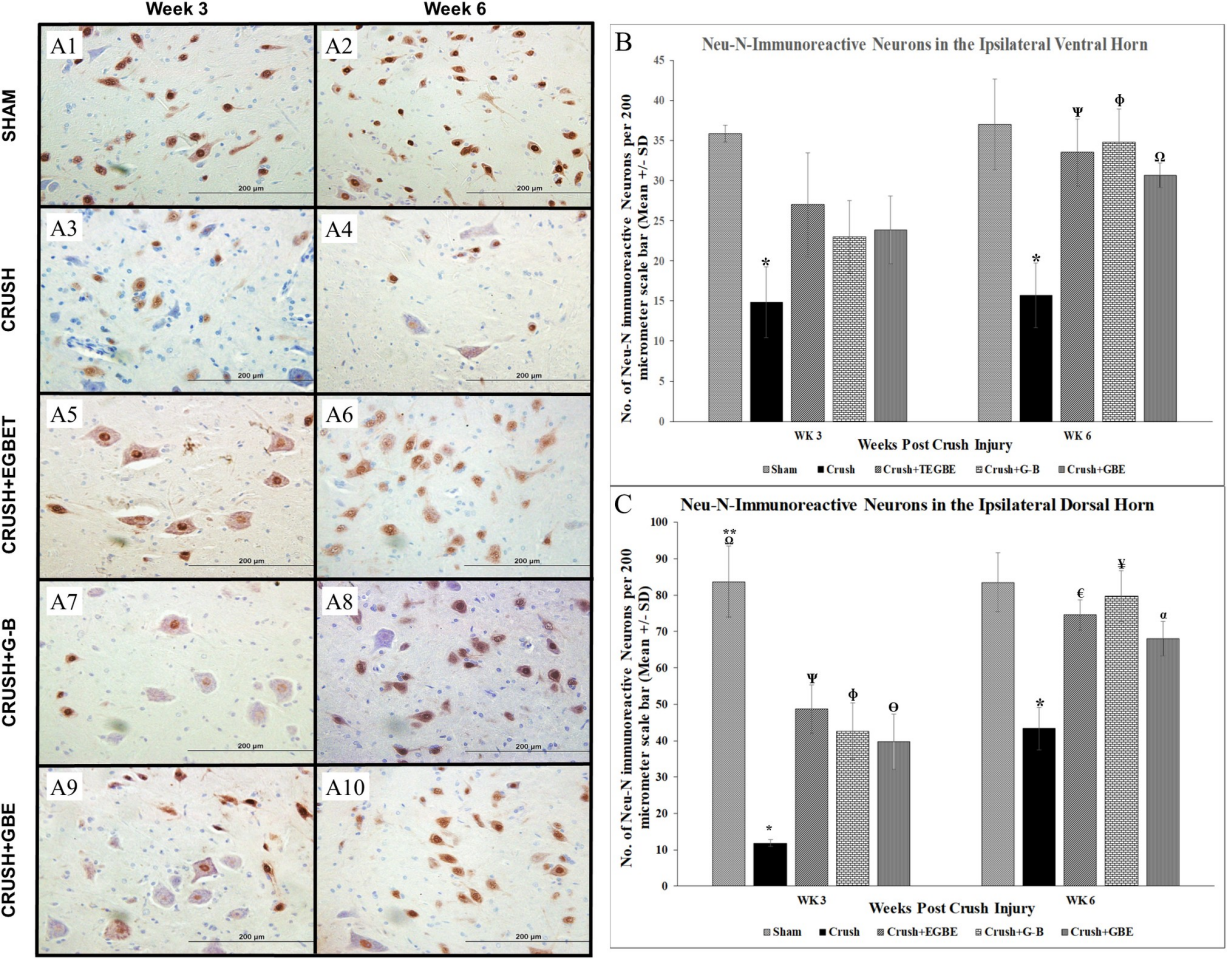
Immunohistochemical analysis. **A:** Representative photomicrographs (40x objective) of lumbar spinal cord ventral grey horn from rats of all groups immunostained for NeuN (NeuN–Anti-NeuN, Clone A60, Mouse Monoclonal Antibody) at week 3 (A1, A3, A5, A7 and A9) and week 6 (A2, A4, A6, A8 and A10) following nerve crush injury. Note that in the crush group (n = 12) (A3 and A4), there is a remarkable decrease in Neu-N immunoreactive motor neurons compared to sham group (n = 12) (A1 and A2) at week 3 and week 6 post-injury, respectively. However, at week 3 post-injury, the ventral grey horns of the TEGBE (n = 12) (A5), G-B (n = 12) (A7), and GBE (n = 6) (A9)-treated groups show a remarkable increase in the number of Neu-N immunoreactive neurons compared with crush animals (A3 and A4). All the treated groups (A6, A8 and A10) maintained a notable more increase in the number of Neu-N immunoreactive neurons at week 6 post-injury. Scale bar = 200 μm. **B, C:** Bar graphs showing the average number of total Neu-N immunoreactive neurons in the ventral grey horn **(B)** dorsal grey horn **(C)** and obtained from the different experimental groups at week 3 and week 6 following sciatic nerve injury. **B:** Note the significant decrease in the Neu-N immunoreactive neurons in crush group compared to the sham group at both time points. In contrast, TEGBE, G-B and GBE-treated groups displayed a gradual increase in the mean number of immunoreactive neurons compared to crush group starting at week 3. This increase in the treated group became highly significant by week 6 post-injury. * indicates p<0.004, crush vs. sham group. Ѱ indicates p<0.006, TEGBE-treated group vs. crush group. ɸ indicates p<0.004, G-B treated-group vs. crush group. Ω indicates p<0.02, GBE-treated group vs. crush group. **C:** Likewise, note the significant (p<0.0001) decrease in NeuN-immunoreactive neurons in the crush group compared to the sham group at both time points. In contrast, all the treated groups with TEGBE, G-B and GBE showed a significant increase in the Neu-N immunoreactive neurons of the dorsal horn compared to crush group starting at week 3 and continued up to week 6 post-injury. * indicates p<0.0001, crush vs. sham group. ** indicates p<0.005, sham vs. G-B and GBE-treated group. Ω indicates p<0.03, sham vs. TEGBE group. Ѱ indicates p<0.0.002, TEGBE-treated group vs. crush group. ɸ indicates p<0.006, G-B-treated group vs. crush group. ϴ indicates p<0.02, GBE-treated group vs. crush group. ¥ indicates p<0.0001, G-B-treated group vs. crush group. € indicates p<0.003, TEGBE-treated group vs. crush group. α indicates p<0.01, GBE-treated group vs. crush group. Data are expressed as means ± SD (One-way ANOVA, Bonferroni’s multiple comparison tests; n = 6 sections/group).

The findings of Neuronal Nuclei (Neu-N) immunostaining neurons (Fig 10A) in both laminas (dorsal and ventral) revealed a clear difference between crush and sham groups, at weeks 3 and 6 following sciatic nerve injury.

Further, treatments with G-B, TEGBE and GBE showed remarkably higher numbers in Neu-N immunoreactive neurons than those in the crush group. Neu-N immunostaining and quantitative analysis indicate the survival of neurons in the spinal cord laminae in the treated groups as compared to the crush group. Fig 10B and 10C show highly significant (P<0.0001) decrease in neuronal counting in the crush group as compared to the sham group in both laminas. Expectedly, G-B, TEGBE and GBE-treated groups showed significant (P<0.05) higher number in Neu-N immunoreactive neurons in dorsal and ventral horns at both time points as compared to the crush group. Meanwhile, all treated groups showed no significant difference in Neu-N immunoreactivity as compared to the sham group.

The neuronal protective characteristics of GBE in the spinal cord revealed in the current study was in agreement with previous findings [40] that showed the positive influence of GBE on dorsal sensory neurons following peripheral nerve injury. G-B was suggested to be the most effective treatment as a neuronal protective when compared to TEGBE and GBE treatments in the ipsilateral spinal cord region based on this analysis (Fig 10B and 10C). It was suggested that the neuroprotective effects of G-B may be due to its potent PAF antagonistic activity in the CNS, and this may contribute to the current findings of the Neu-N analysis [41]. Further, this study demonstrated, for the first time, the positive effects of G-B, TEGBE and GBE on the quantitative analysis of Neu-N immunostained spinal cord neurons post sciatic crush injury.

**Fig 11:**
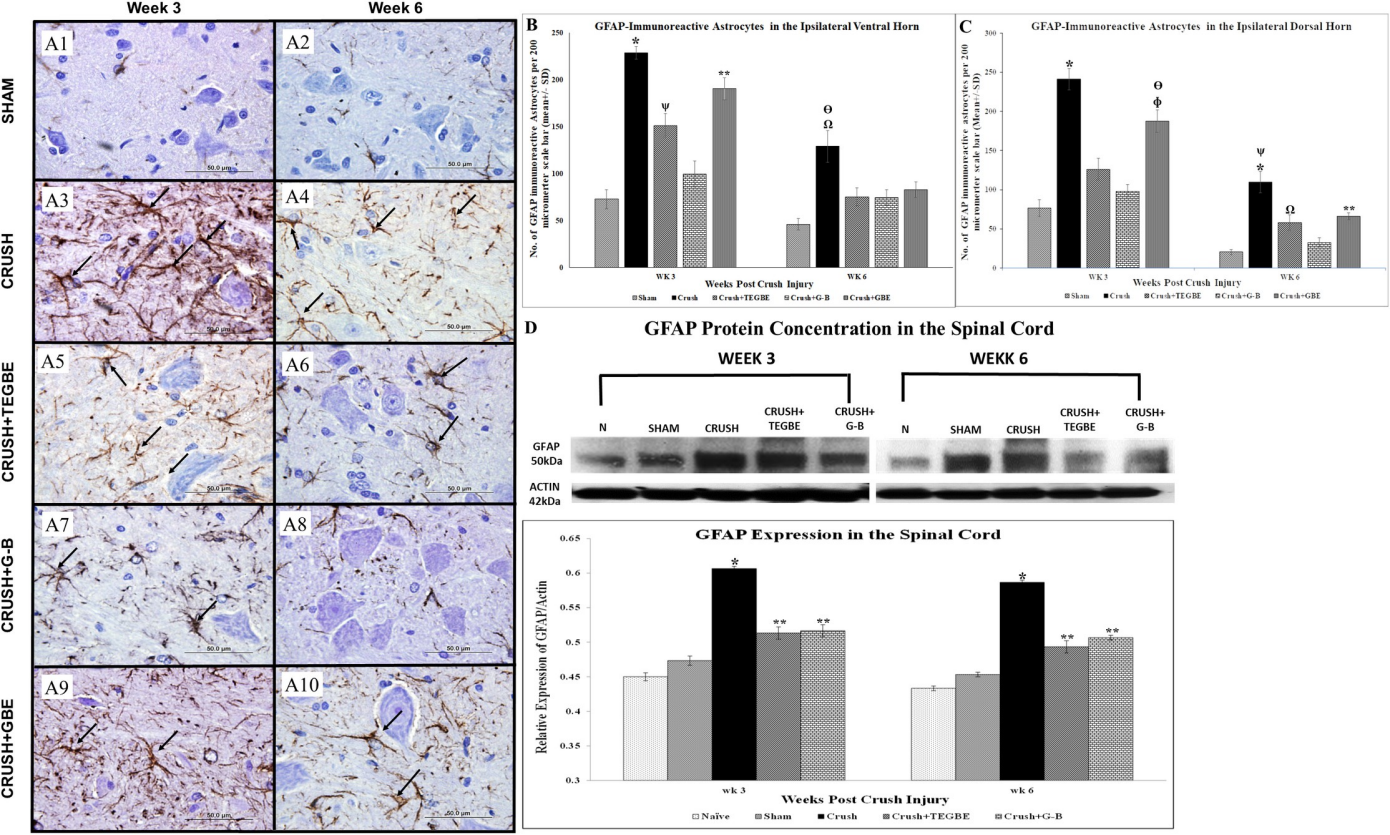
Spinal cord staining with GFAP. **A:** Representative photomicrographs (100x objective) of the ipsilateral ventral grey horn of L3-L6 spinal cords sections from all groups immunostained for GFAP (GFAP (GA-5): sc-58766, Mouse Monoclonal–Santa Cruz Biotechnology) at week 3 **(A1, A3, A5, A7 and A9)** and week 6 **(A2, A4, A6, A8 and A10)** following nerve crush injury. The sham group (n = 12) (A1 and A2) show normal and clear GFAP-immunoreactive astrocytes in the ventral horn. Whereas, the crush group (n = 12) (A3 and A4) display a remarkable increase in GFAP immunoreactive astrocytes. The TEGBE (n = 12) **(A5, A6)**, G-B (n = 12) **(A7, A8)** and GBE-treated (n = 6) **(A9, A10)** groups show an observable decrease in the GFAP-immunoreactive astrocytes compared to crush at the week 3 and week 6 post-injury. Black arrows indicate the GFAP immunoreactive astrocytes. Scale bar = 200 μm. **B, C:** Bar graphs showing the average number of total GFAP immunoreactive neurons in the ventral grey horn (**B**) dorsal grey horn (**C**) and obtained from the different experimental groups at week 3 and week 6 following sciatic nerve injury. Data show a significant decrease in the number of the GFAP-immunoreactive astrocytes in the ventral and dorsal grey matters at week 3 and week 6 post-injury. **B:** Note the significant increase in the GFAP immunoreactive astrocytes in the ventral horn in crush compared to sham and treated groups at week 3 and week 6 post-injury. * indicates p<0.0001, crush group vs. sham group, TEGBE and G-B treated groups. ** indicates p<0.0001, GBE treated group vs. sham group and G-B-treated group. Ψ indicates p<0.002, TEGBE-treated group vs. sham group and G-B-treated group. Ω indicates p<0.0001, crush vs. sham group. ϴ indicates p<0.005, crush vs. all treated groups. **C:** Note the significant increase in GFAP immunoreactive astrocytes in the dorsal horn in crush compared to sham, TEGBE and G-B and GBE-treated groups at week 3 and week 6 post-surgery. * indicates p<0.0001, crush vs. sham group, TEGBE and G-B-treated groups. ψ indicates p<0.02, crush compared to GBE treated group. ɸ indicates p<0.0001, GBE treated group vs. sham group and G-B-treated group. ϴ indicates p<0.02, GBE-treated group vs. TEGBE-treated group. ** indicates p<0.009, GBE-treated group vs. sham group. Ω indicates p<0.05, TEGBE-treated group vs. sham group. Data represent mean ± SD (n = 6 /group). **D:** Illustrates the Western blot graph and the statistical data analysis of the GFAP expression in the spinal cords obtained from all the experimental groups except GBE treated group due to a shortage of animal supply. In each group, the lumbar spinal cords from three rats were analyzed at week 3 and week 6 post-injury. The antibody used to detect the GFAP (Anti-GFAP antibody (ab7260)–Abcam Biochemical®, Cambridge, MA, USA). The bar graph shows the GFAP density in spinal cord samples (n = 3 rats/group) normalized with densities of actin band (Molecular weight-42). The labeled GFAP bands were quantified. Note that the crush group exhibits a significant (*p<0.0001) increase in the GFAP expression compared to sham and naive (n = 6) groups. Whereas the TEGBE and G-B treated groups show a substantial decrease in GFAP immunoblotting, but still significantly (**p<0.001) higher compared to naive and sham animals at weeks 3 and 6 post-injury.

Immunohistochemical analysis (IHC) of glial fibrillary acid protein (GFAP) indicated the state of astrocytes and glial cells [42]. Staining the spinal cord sections with GFAP (Fig 11A1–11A10) revealed a remarkable increase in astrocytes activity in the crush group among other groups.

Data analysis of GFAP (Fig 11B and 11C) revealed a significant (*P*<0.0001) increase in GFAP immunostained astrocytes in the crush as compared to the sham group in both laminas at both time points following sciatic nerve injury. The reactive astrocytes and gliosis, in the crush group, and the increased GFAP-immunoreactive distribution was similar to astrocyte hypertrophy observed in previous studies [43]. However, in the sham group, the astrocytes’ reactivity appeared to be completely quiescent, as consistent with a previous study [44]. Unlike, the crush group, the treated groups with GB and TEGBE showed a significant (P<0.0.001) decrease in GFAP immunostained neurons in the dorsal horn (Fig 11C). G-B-treated group showed no significant change (*p*>0.05) in the GFAP immunoreactive astrocytes intensity as compared to the sham rats in the dorsal and ventral horns at week 3 and week 6 post-injury. On the other hand, TEGBE and GBE-treated groups showed mixed results in the calculated mean values. The inhibition of glial hypertrophy in the spinal cord, by G-B, following sciatic nerve injury might be attributed to its neuroprotective mechanisms due to PAF antagonistic activity, which prevented the GFAP immunoreactivity in astrocytes [41].

Moreover, western blotting data for GFAP immunoreactivity (Fig 11D) supported the significant (P<0.0001) increase in astrocytes activity in the crush group as compared to the other groups. However, G-B and TEGBE-treated groups showed a substantial decrease in GFAP immunoblotting, but still significantly (*P*<0.001) higher than those of the naive and sham animals at weeks 3 and 6 post-injury. The protective effect of GBE on astrocytes of rat hippocampus was shown previously [45].

Further, immunohistological findings, reported before [46], showed the neuroprotection of GBE in a model of vascular dementia, which was accompanied by a reduction in the astrogliosis and a decrease in GFAP immunoreactivity. However, there were no reported studies on the effects of GBE on GFAP immunoreactivity in the spinal cord post crush sciatic injury.

**Fig 12:**
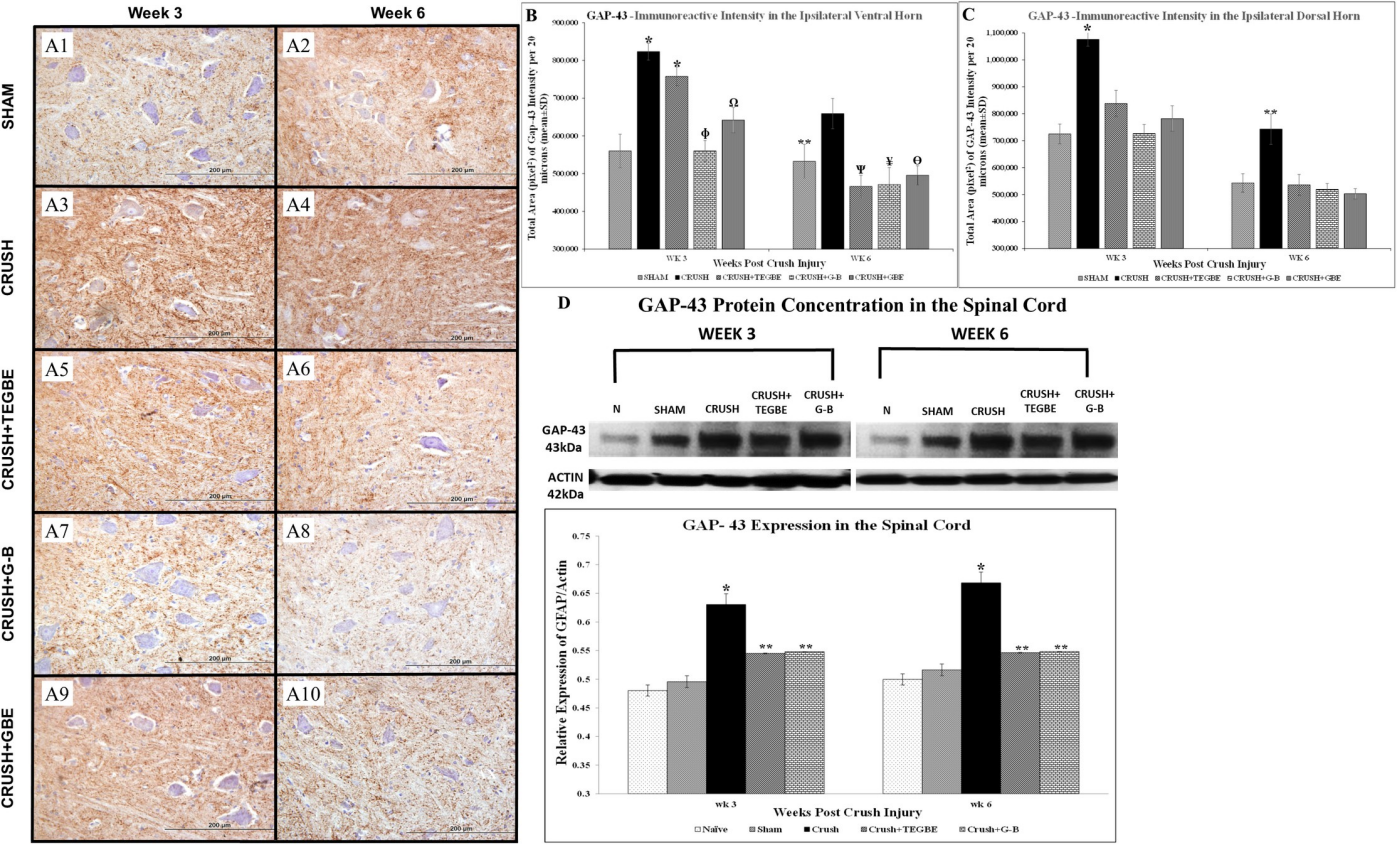
GAP-43 immunoreactivity. **A:** Representative photomicrographs (40x objective) of ipsilateral ventral grey horn of L3-L6 spinal cords sections from all groups immunostained for GAP-43 (GAP-43 Antibody (B-5): sc-17790 –Santa Cruz Biotechnology) at week 3 **(A1, A3, A5, A7 and A9)** and week 6 **(A2, A4, A6, A8 and A10)** following nerve crush injury. Note that the crush group (n = 12) (**A3, A4**) display a remarkable increase in GAP-43 immunoreactivity when compared to sham (n = 12) (**A1, A2**) group at week 3 and week 6 post-injury. Whereas treated animals with TEGBE (n = 12) (**A5, A6**), G-B (n = 12) (**A7, A8**) and GBE (n = 6) (**A9, A10**) show a prominent decrease in GAP-43 immunoreactivity in the ventral horn compared to sham group at week 3 and week 6 following sciatic nerve injury. Scale bar = 200 μm. **B, C:** Bar graphs showing the average GAP-43 immunoreactive areas in the ventral grey horn **(B)** dorsal grey horn (**C**) of the spinal cord and obtained from the different experimental groups at week 3 and week 6 following sciatic nerve injury. Note that the significant increase in the GAP-43 immunoreactive intensities in the ventral **(B)** and dorsal **(C)** horns of the crush animals compared to sham group at week 3 and week 6 post-injury. Whereas the EGBE, G-B and GBE-treated groups showed significant decrease intensity of the GAP-43 immunoreactivity at week 3 and week 6 compared to crush group. **B:** * indicates p<0.0001, crush group and TEGBE-treated group vs. sham group. ɸ indicates p<0.0001, G-B-treated group vs. crush group and TEGBE-treated group. Ω indicates p<0.005, GBE-treated group vs. crush group. ** indicates p<0.03, sham vs. CRUSH = group. Ѱ indicates p<0.002, TEGBE-treated group vs. crush group. ¥ indicates p<0.003, G-B-treated group vs. crush group. ϴ indicates p<0.006, GBE-treated group vs. crush group. **C:** * indicates P<0.0001, crush vs. sham and all treated groups. ** indicates p<0.002, crush vs. sham and all treated groups. The intensity area in pixel2 was measured using Cell-Sens software of 20x magnification of all sections (n = 6 /group). Data represent mean ± SEM. Scale bar = 20 μm. **D:** Illustrates the Western blot graph and the statistical data analysis of the GAP-43 expression in the spinal cords obtained from the experimental groups. In each group, the lumbar spinal cords from three rats were analyzed at week 3 and week 6 post-injury. The bar graph shows the GAP-43 immunoreactive intensities in spinal cord samples (n = 3 rats/group) normalized with densities of actin band (Molecular weight-42). The labeled GAP-43 bands were quantified. Note that the significant (*p<0.0001) increase in the GAP-43 expression is in the crush group compared to all groups at week 3 and week 6 post nerve injury. Whereas the TEGBE and G-B treatments induced a substantial decrease in GFAP immunoblotting, but still significantly (**p<0.05) higher compared to naive animals (n = 6) at weeks 3 and 6 post-injury.

Growth-associated proteins-43 (GAP-43) is a membrane protein involved in neuronal development and plasticity. It is also considered an essential factor for proper neuronal regeneration, and an integral component of the axotomy response of primary sensory neurons [47]. In addition to IHC tests, GAP-43 immunoreactivity photomicrographs (Fig 12A1–12A10) showed ubiquitously high expression of axonal sprouting in the crush group, unlike the treated groups, in both ventral and dorsal horns. GAP-43 data analysis (Fig 12B and 12C), showed significant (P<0.001) increase in GAP-43 intensity in the crush group as compared to sham group at both time points.

A previous study [48] supported the current data that GAP-43 immunoreactivity increased in a sciatic crush injury model indicating an active attempt by the injured spinal cord neurons to repair or protect themselves from the crush injury insult. This was supported by western blotting data analysis of GAP-43 immunoreactivity (Fig 12D) which showed a highly significant (*P*<0.0001) increase in GAP-43 expression in the crush group as compared to other groups. In contrast, G-B treatment successfully maintained the levels of GAP-43 in both the dorsal and ventral horns as compared to the sham group (*p*>0.05) at week 3 and week 6 following crush injury. Likewise, TEGBE and GBE-treated groups showed mixed results, however, a significant (*P*<0.01) decrease in GAP-43 intensity at week 6 in both dorsal and ventral horns was noticeable as compared to the crush group.

Furthermore, it was revealed that TEGBE and G-B treatments induced a substantial decrease in the GAP-43 immunoblotting but still significantly (*P*<0.05) higher as compared to naive animals at weeks 3 and 6 post-injury. The decrease in GAP-43 immunoreactivity in the current findings by GBE, TEGBE and G-B was in full agreement with previous studies, thus indicating the central prevention of axonal sprouting following sciatic crush injury [49]. Further, the GAP-43 decrease may reveal that the spinal neurons are well protected by the treatments and survived the crush injury insult. The intensity of the GAP-43 immunoreactivity was highly ameliorated by G-B more than GBE and TEGBE treatments, indicating a more potent central neuroprotective effects for G-B.

To date, there were no reported studies on the GAP-43 immunohistological analysis conducted following the administration of G-B, TEGBE, or GBE after sciatic nerve crush injury.

The current investigations of IHC were consistent with previous studies examined the neuroprotective properties of GBE on spinal cord neurons both *in vitro* and *in vivo*, [50,51] and neurogenerative diseases, such as Alzheimer’s and Parkinson’s diseases [52].

Although GBE and TEGBE are shown to be effective in the recovery of the sciatic nerve following crush injury, G-B was shown to be the most effective treatment. Such evidence might be due to the peculiar pharmacokinetic properties of G-B, as it was reported to cross the blood-brain barrier in an active form and at adequate concentrations when compared to other ginkgolides [53]. Pharmacokinetic studies showed that ginkgolide C concentration in plasma is negligible. Additionally, the effects of the terpene trilactones were shown to be due to only ginkgolides A and B and bilobalide [54]. Furthermore, another study [55] reported that not all flavonoids were centrally absorbed.

Therefore, the reasons that GBE was less effective in protecting the spinal cord neurons as compared to the terpene trilactones-enriched extract (TEGBE) and the isolated pure G-B may be due to its poor absorption into the CNS.

In conclusion, this study presented a strong evidence for GBE and its ginkgolides, in particular G-B, on the early recovery of the crushed nerve, neuronal protection, and modulation of the histopathological alterations of the spinal cord milieu in the sciatic crush nerve rat model.

Nonetheless, further experiments should be performed to clarify the mechanism of action of GBE, G-B, as well as other ginkgolides. Future investigations might be required to study the role of GBE and G-B on Schwann cells and neurotrophic factors. Also, experiments should be planned to compare G-B or other ginkgolides, or even GBE, with an approved effective standard therapy on crush sciatic nerve injury model, such as vitamin B complex.

## Supporting information

S1 Fig ^13^C NMR (150 MHz, CD_3_OD) spectrum of DGA-I-81D. (PDF)

S2 Fig DEPT 135° spectrum of DGA-I-81D. (PDF)

S3 Fig HSQC spectrum of DGA-I-81D. (PDF)

S4 Fig COSY spectrum of DGA-I-81D. (PDF)

S5 Fig ^1^H NMR (600 MHz, CD_3_OD) spectrum of DGA-I-81D. (PDF)

S6 Fig HMBC spectrum of DGA-I-81D. (PDF)

S7 Fig Electron microscopy. A: Sciatic nerve electron micrographs from the different experimental groups at week 3 and week 6 post-injury (5000x). Naïve (n = 6) **(A and B)** and sham (n = 12) **(C and D)** animals show the normal nerve fibers and healthy appearance of myelin sheaths (M) and axons (AX) at week 3 and week 6, respectively. The crush **(E)** group (n = 12), at week 3, exhibits very irregular shaped myelin sheaths and axonal fibers along with disintegrated and remnants of myelin scattered in between the axons (arrows). At week 6, the majority of the axons and nerve fibers in the crush group **(F)** are still small in size and thinly myelinated. Also, the crush nerves exhibit newly regenerated nerve fibers (circles) along with unmyelinated axonal fibers and disintegrated myelin sheaths (small arrow). The TEGBE (n = 12) **(G and H)**, G-B (n = 12) **(I and J)** and GBE-treated (n = 6) **(K and L)** groups show normal nerve fibers with healthy appearance myelin sheaths and collagen fibers (CF) compared to **(D and F)**. Note that at week 6 the all treated groups **(H, J and L)** displayed more remarkable intact and organized extracellular matrix in addition to normal and healthy myelin sheaths in addition to newly regenerated axonal fibers (circles).
(TIF)
