# Supplementary material for: Neurotherapeutic effects of Ginkgo biloba extract and its terpene trilactone, ginkgolide B, on sciatic crush injury model: A new evidence
Source: PLoS One. 2019 Dec 26;14(12):e0226626. doi: 10.1371/journal.pone.0226626 (PMC6932810; doi:10.1371/journal.pone.0226626)
Supplement: S1 Fig — (PDF) [file pone.0226626.s001.pdf]

S1 Fig

13C decoupled spectra Dr.Orabi GL-B in MEOD

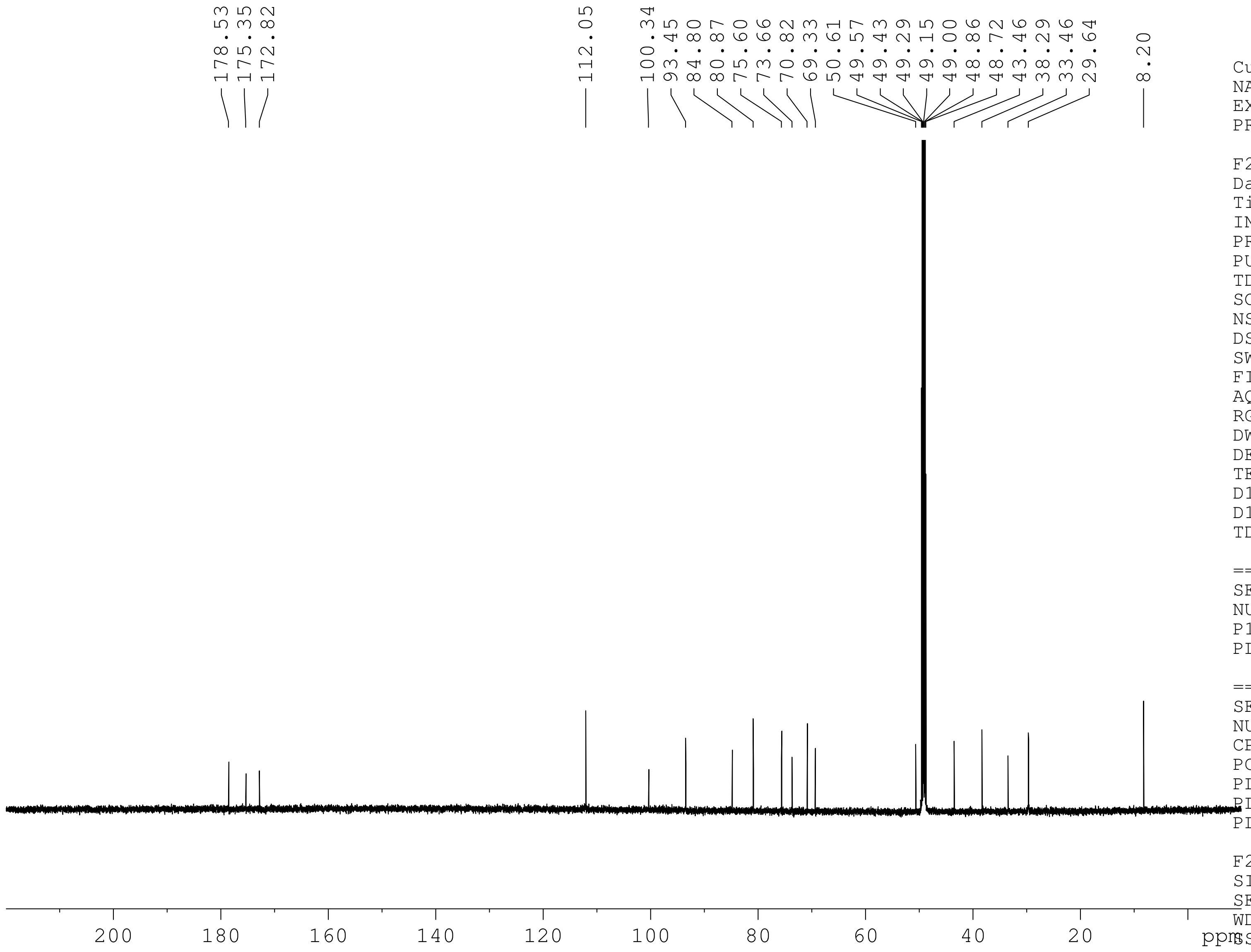

BRUKER

Current Data Parameters

NAME	GL-B
EXPNO	2
PROCNO	1

F2 - Acquisition Parameters

Date_	20160317
Time	12.14
INSTRUM	spect
PROBHD	5 mm PABBO BB-
PULPROG	zgpg30
TD	65536
SOLVENT	MeOD
NS	310
DS	4
SWH	36057.691 Hz
FIDRES	0.550197 Hz
AQ	0.9087659 sec
RG	203
DW	13.867 usec
DE	50.00 usec
TE	298.0 K
D1	2.00000000 sec
D11	0.03000000 sec
TD0	1

======== CHANNEL f1 ========

SFO1	150.9178979 MHz
NUC1	13C
P1	8.80 usec
PLW1	78.13500214 W

======== CHANNEL f2 ========

SFO2	600.1324005 MHz
NUC2	1H
CPDPRG[2	waltz65
PCPD2	70.00 usec
PLW2	27.82500076 W
PLW12	0.63804001 W
PLW13	0.31264001 W

F2 - Processing parameters

SI	32768
SF	150.9025745 MHz
WDW	EM
SSB	0
LB	1.00 Hz
GB	0
PC	1.40

ANALYST: RAJANI KRISHNA PILLAI
*Please acknowledge RSPU Project GS01/03 in your publications.
*Please visit our website www.science.saf.kuniv.edu for more information on RSPU facilities.
*Please collect your samples within one week after receiving the results.
