# Supplementary material for: Neurotherapeutic effects of Ginkgo biloba extract and its terpene trilactone, ginkgolide B, on sciatic crush injury model: A new evidence
Source: PLoS One. 2019 Dec 26;14(12):e0226626. doi: 10.1371/journal.pone.0226626 (PMC6932810; doi:10.1371/journal.pone.0226626)
Supplement: S2 Fig — (PDF) [file pone.0226626.s002.pdf]

S2 Fig

13C DEPT 135 spectra Dr.Orabi GL-B in MEOD

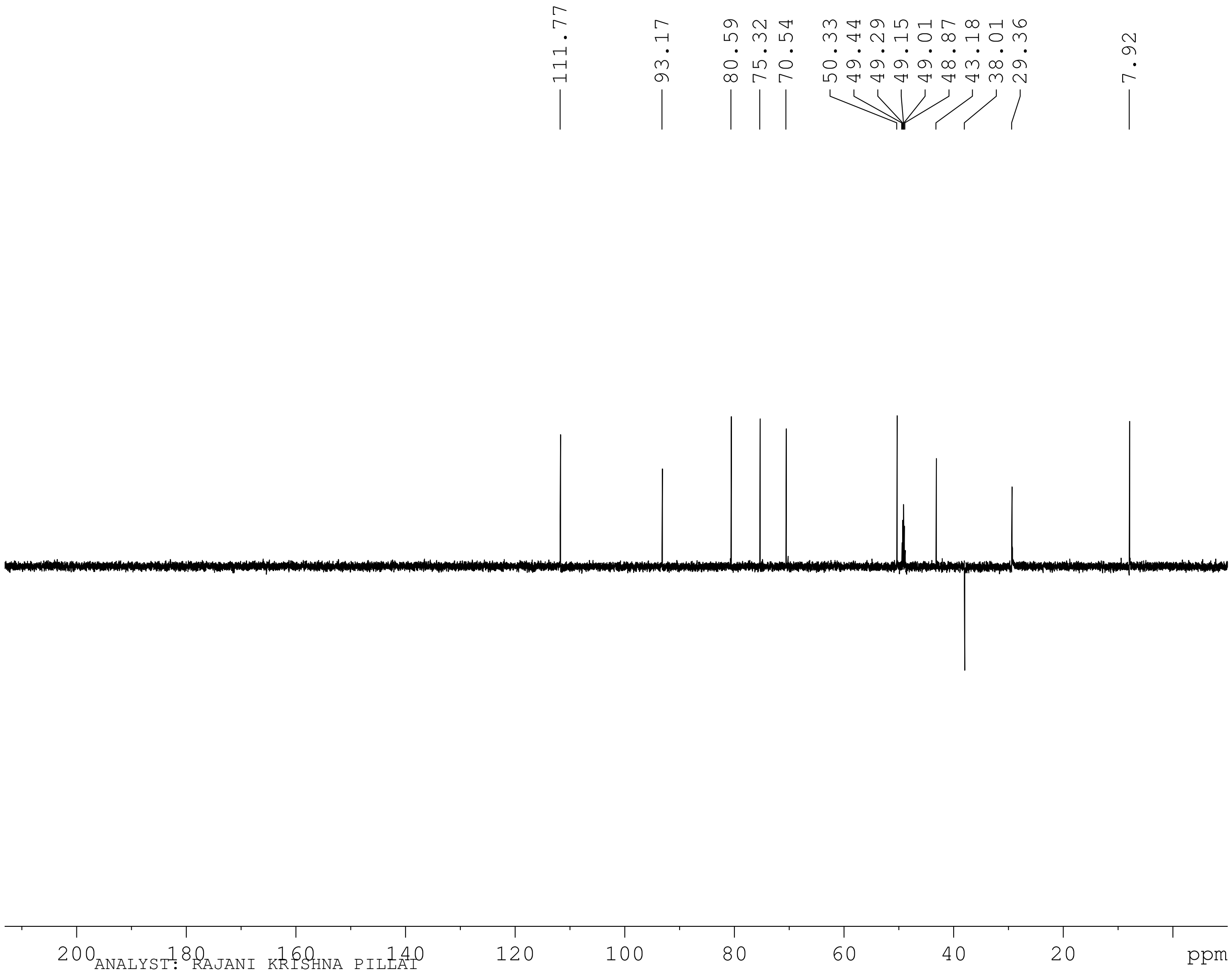

ANALYST: RAJANI KRISHNA PILLAI
*Please acknowledge RSPU Project GS01/03 in your publications.
*Please visit our website www.science.saf.kuniv.edu for more information on RSPU facilities.
*Please collect your samples within one week after receiving the results.

Current Data Parameters
NAME GL-B
EXPNO 5
PROCNO 1

F2 - Acquisition Parameters
Date_ 20160317
Time 12.51
INSTRUM spect
PROBHD 5 mm PABBO BB-
PULPROG deptsp135
TD 65536
SOLVENT MeOD
NS 200
DS 4
SWH 36057.691 Hz
FIDRES 0.550197 Hz
AQ 0.9087659 sec
RG 203
DW 13.867 usec
DE 50.00 usec
TE 298.0 K
CNST2 145.0000000
D1 2.00000000 sec
D2 0.00344828 sec
D12 0.00002000 sec
TD0 1

======== CHANNEL f1 ========
SFO1 150.9178979 MHz
NUC1 13C
P1 8.80 usec
P13 2000.00 usec
PLW0 0 W
PLW1 78.13500214 W
SPNAM[5] Crp60comp.4
SPOAL5 0.500
SPOFFS5 0 Hz
SPW5 9.24489975 W

======== CHANNEL f2 ========
SFO2 600.1324005 MHz
NUC2 1H
CPDPRG[2 waltz65
P3 10.60 usec
P4 21.20 usec
PCPD2 70.00 usec
PLW2 27.82500076 W
PLW12 0.63804001 W

F2 - Processing parameters
SI 32768
SF 150.9026167 MHz
WDW EM
SSB 0
LB 1.00 Hz
GB 0
PC 1.40
