# Supplementary material for: Neurotherapeutic effects of Ginkgo biloba extract and its terpene trilactone, ginkgolide B, on sciatic crush injury model: A new evidence
Source: PLoS One. 2019 Dec 26;14(12):e0226626. doi: 10.1371/journal.pone.0226626 (PMC6932810; doi:10.1371/journal.pone.0226626)
Supplement: S3 Fig — (PDF) [file pone.0226626.s003.pdf]

S3 Fig

COSYspectrum Dr.Orabi GL-B in MeOD

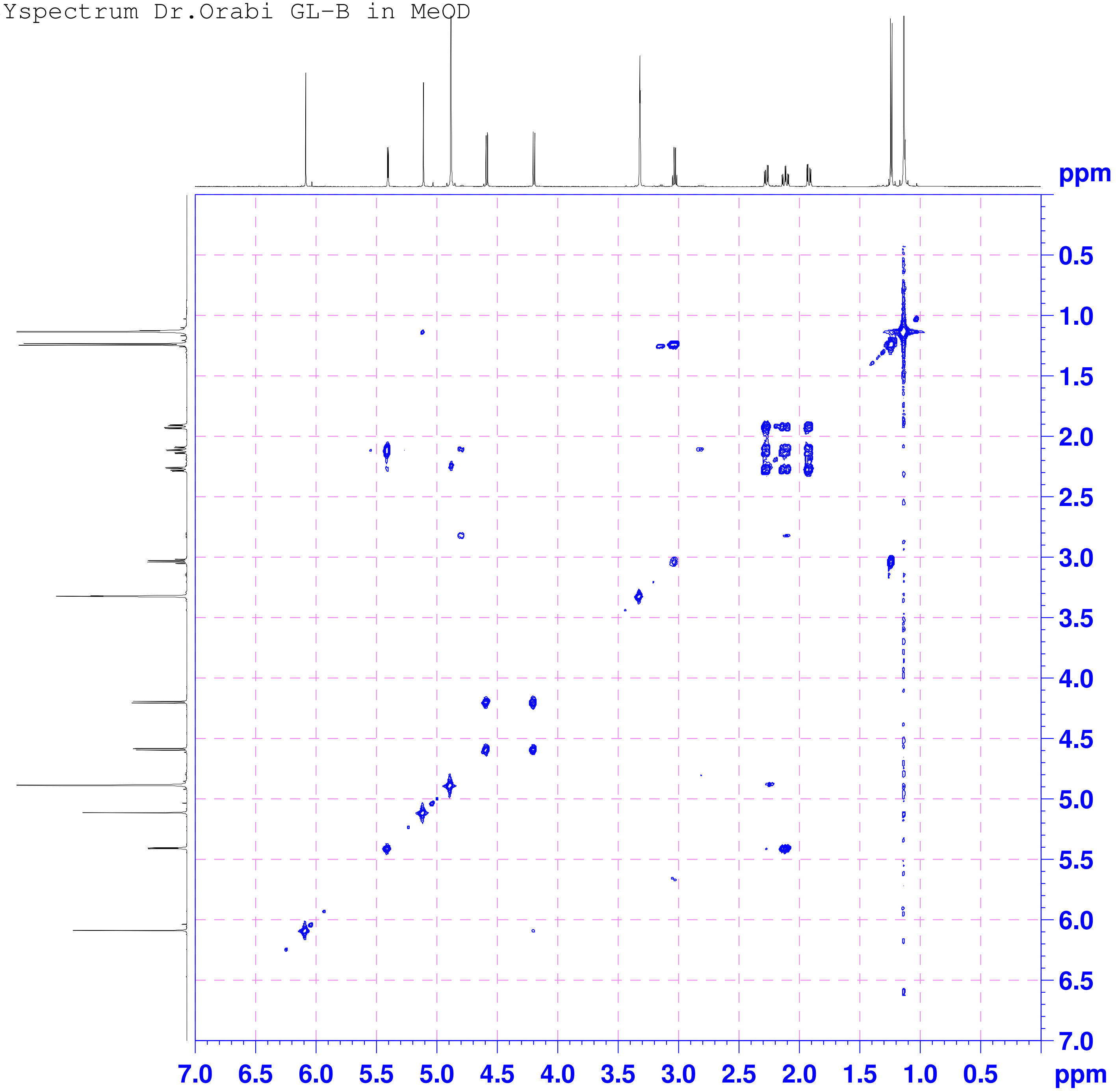

Current Data Parameters	
NAME	GL-B
EXPNO	10
PROCNO	1
F2 - Acquisition Parameters	
Date_	20160324
Time	1.13
INSTRUM	spect
PROBHD	5 mm PABBO BB-
PULPROG	cosygpppqf
TD	2048
SOLVENT	MeOD
NS	32
DS	8
SWH	3731.343 Hz
FIDRES	1.821945 Hz
AQ	0.2744320 sec
RG	90.5
DW	134.000 usec
DE	20.00 usec
TE	295.3 K
D0	0.00000300 sec
D1	0.50014442 sec
D11	0.03000000 sec
D12	0.00002000 sec
D13	0.00000400 sec
D16	0.00020000 sec
IN0	0.00026800 sec
======== CHANNEL f1 ========	
SFO1	600.1321123 MHz
NUC1	1H
P0	10.60 usec
P1	10.60 usec
P17	2500.00 usec
PLW1	27.82500076 W
PLW10	4.62489986 W
====== GRADIENT CHANNEL =====	
GPNAM[1]	SINE.100
GPZ1	20.00 %
P16	1000.00 usec
F1 - Acquisition parameters	
TD	320
SFO1	600.1321 MHz
FIDRES	11.660448 Hz
SW	6.218 ppm
FnMODE	QF
F2 - Processing parameters	
SI	2048
SF	600.1299969 MHz
WDW	SINE
SSB	0
LB	0 Hz
GB	0
PC	1.40
F1 - Processing parameters	
SI	2048
MC2	QF
SF	600.1299965 MHz
WDW	SINE
SSB	0
LB	0 Hz
GB	0
