# Supplementary material for: Neurotherapeutic effects of Ginkgo biloba extract and its terpene trilactone, ginkgolide B, on sciatic crush injury model: A new evidence
Source: PLoS One. 2019 Dec 26;14(12):e0226626. doi: 10.1371/journal.pone.0226626 (PMC6932810; doi:10.1371/journal.pone.0226626)
Supplement: S4 Fig — (PDF) [file pone.0226626.s004.pdf]

S4 Fig

13C HSQC spectrum Dr.Orabi GL-B in MeOD

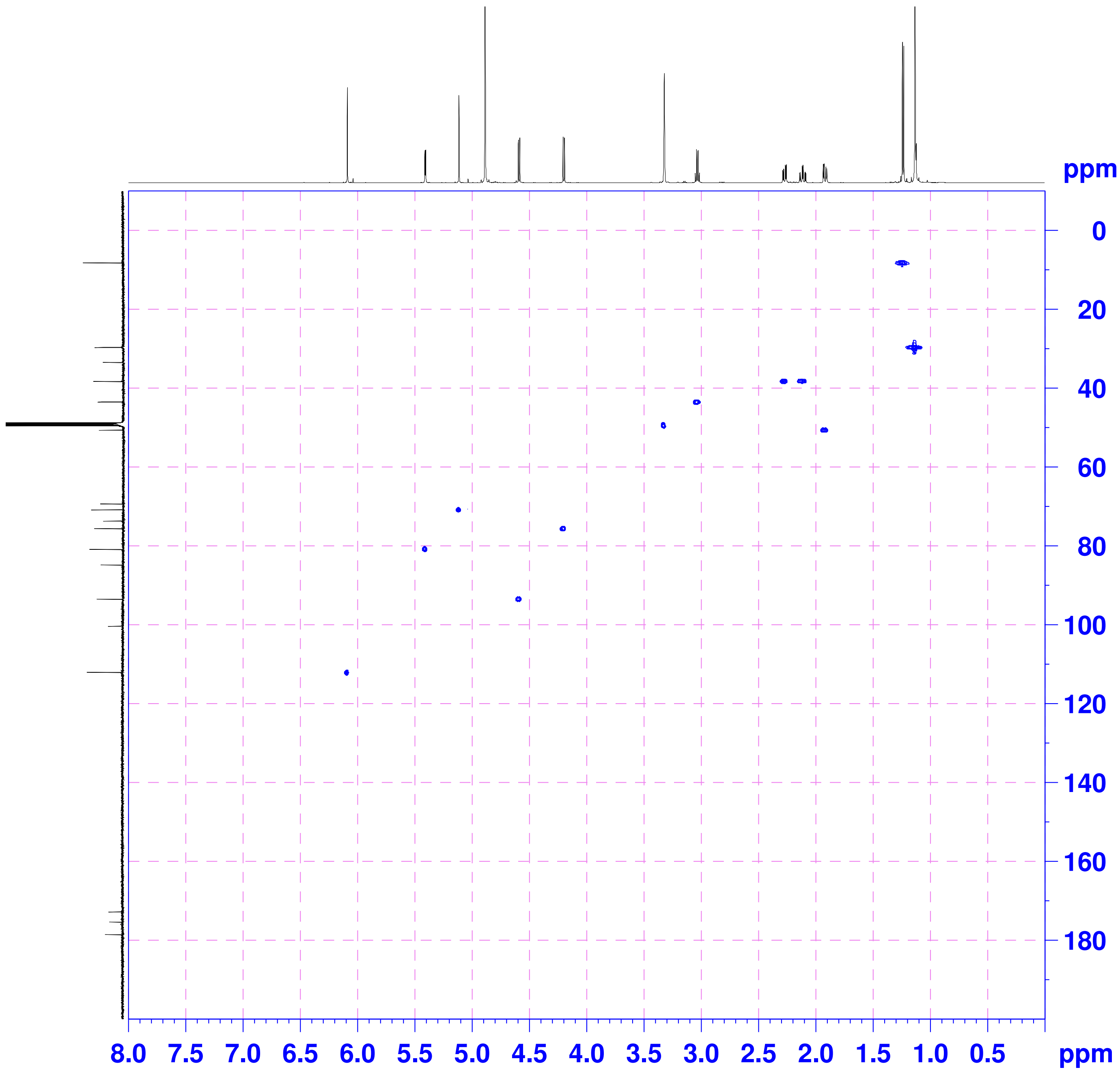


```
Current Data Parameters
NAME                  GL-B
EXPNO                   11
PROCNO                   1

F2 - Acquisition Parameters
Date_             20160324
Time                  3.42
INSTRUM              spect
PROBHD   5 mm PABBO BB-
PULPROG     hsqcetgpsp.2
TD                    2048
SOLVENT               MeOD
NS                      32
DS                      16
SWH               3731.343 Hz
FIDRES            1.821945 Hz
AQ               0.2744320 sec
RG                     203
DW                 134.000 usec
DE                   20.00 usec
TE                   295.8 K
CNST2          145.0000000
D0              0.00000300 sec
D1              1.81076503 sec
D4              0.00172414 sec
D11             0.03000000 sec
D16             0.00020000 sec
IN0             0.00001510 sec
ZGOPTNS

======== CHANNEL f1 ========
SFO1         600.1321123 MHz
NUC1                    1H
P1                   10.60 usec
P2                   21.20 usec
P28                1000.00 usec
PLW1           27.82500076 W

======== CHANNEL f2 ========
SFO2         150.9178993 MHz
NUC2                   13C
CPDPRG[2             garp4
P3                    8.80 usec
P14                 500.00 usec
P24                2000.00 usec
PCPD2                60.00 usec
PLW0      0 W
PLW2           78.13500214 W
PLW12           1.68079996 W
SPNAM[3] Crp60,0.5,20.1
SPOAL3               0.500
SPOFFS3   0 Hz
SPW3            9.24489975 W
SPNAM[7]      Crp60comp.4
SPOAL7               0.500
SPOFFS7   0 Hz
SPW7            9.24489975 W

====== GRADIENT CHANNEL =====
GPNAM[1]          SINE.100
GPNAM[2]          SINE.100
GPZ1                 80.00 %
GPZ2                 20.10 %
P16                1000.00 usec

F1 - Acquisition parameters
TD                     341
SFO1              150.9179 MHz
FIDRES           97.104347 Hz
SW                 219.408 ppm
FnMODE        Echo-Antiecho

F2 - Processing parameters
SI                    2048
SF             600.1299963 MHz
WDW                  QSINE
SSB                      2
LB        0 Hz
GB        0
PC                    1.40

F1 - Processing parameters
SI                    1024
MC2           echo-antiecho
SF             150.9025526 MHz
WDW                  QSINE
SSB                      2
LB        0 Hz
GB        0
```
