# Supplementary material for: Neurotherapeutic effects of Ginkgo biloba extract and its terpene trilactone, ginkgolide B, on sciatic crush injury model: A new evidence
Source: PLoS One. 2019 Dec 26;14(12):e0226626. doi: 10.1371/journal.pone.0226626 (PMC6932810; doi:10.1371/journal.pone.0226626)
Supplement: S5 Fig — (PDF) [file pone.0226626.s005.pdf]

1H spectra Dr.Orabi GL-B in MEOD

S5 Fig

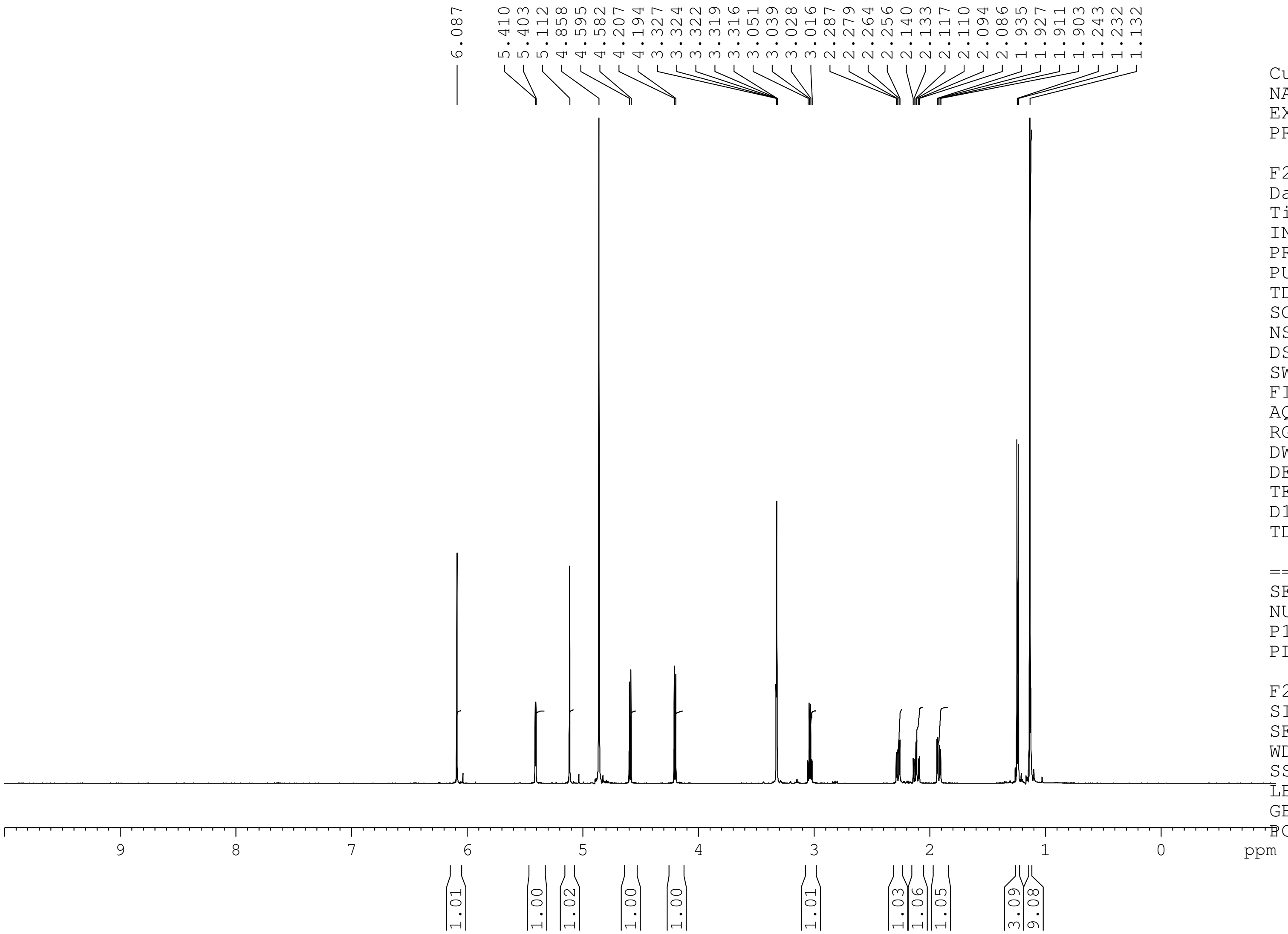

Current Data Parameters

NAME	GL-B
EXPNO	1
PROCNO	1

F2 - Acquisition Parameters

Date_	20160317
Time	12.05
INSTRUM	spect
PROBHD	5 mm PABBO BB-
PULPROG	zg30
TD	65536
SOLVENT	MeOD
NS	8
DS	2
SWH	12335.526 Hz
FIDRES	0.188225 Hz
AQ	2.6563926 sec
RG	203
DW	40.533 usec
DE	20.00 usec
TE	298.0 K
D1	1.00000000 sec
TD0	1

======== CHANNEL f1 ========

SFO1	600.1337060 MHz
NUC1	1H
P1	10.60 usec
PLW1	27.82500076 W

F2 - Processing parameters

SI	32768
SF	600.1300000 MHz
WDW	EM
SSB	0
LB	0.30 Hz
GB	0
PC	1.00

ANALYST: RAJANI KRISHNA PILLAI

*Please acknowledge RSPU Project GS01/03 in your publications.
*Please visit our website www.science.saf.kuniv.edu for more information on RSPU facilities.
*Please collect your samples within one week after receiving the results.
