# Supplementary material for: Neurotherapeutic effects of Ginkgo biloba extract and its terpene trilactone, ginkgolide B, on sciatic crush injury model: A new evidence
Source: PLoS One. 2019 Dec 26;14(12):e0226626. doi: 10.1371/journal.pone.0226626 (PMC6932810; doi:10.1371/journal.pone.0226626)
Supplement: S6 Fig — (PDF) [file pone.0226626.s006.pdf]

S6 Fig

13C HMBC spectrum Dr.Orabi GL-B in MeOD

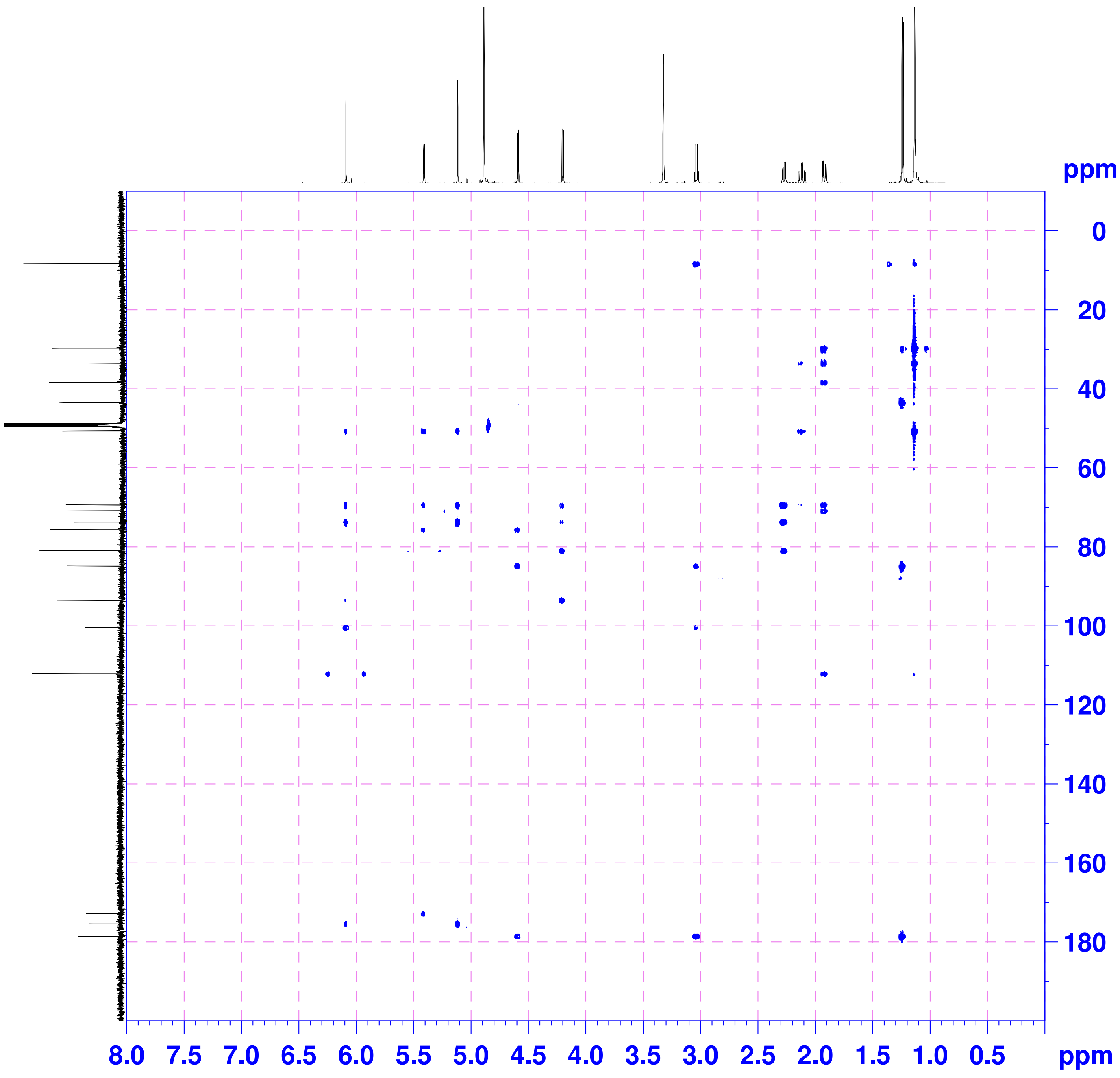

Current Data Parameters
NAME GL-B
EXPNO 12
PROCNO 1

F2 - Acquisition Parameters
Date_ 20160325
Time 3.32
INSTRUM spect
PROBHD 5 mm PABBO BB-
PULPROG hmbcetgpl2nd
TD 2048
SOLVENT MeOD
NS 32
DS 16
SWH 3731.343 Hz
FIDRES 1.821945 Hz
AQ 0.2744320 sec
RG 203
DW 134.000 usec
DE 20.00 usec
TE 300.1 K
CNST6 125.0000000
CNST7 165.0000000
CNST13 10.0000000
CNST30 0.5981150
D0 0.00000300 sec
D1 1.31076503 sec
D6 0.05000000 sec
D16 0.00020000 sec
IN0 0.00001490 sec

======== CHANNEL f1 ========
SFO1 600.1321123 MHz
NUC1 1H
P1 10.60 usec
P2 21.20 usec
PLW1 27.82500076 W

======== CHANNEL f2 ========
SFO2 150.9178738 MHz
NUC2 13C
P3 8.80 usec
P24 2000.00 usec
PLW2 78.13500214 W
SPNAM[7] Crp60comp.4
SPOAL7 0.500
SPOFFS7 0 Hz
SPW7 9.24489975 W

====== GRADIENT CHANNEL =====
GPNAM[1] SINE.100
GPNAM[3] SINE.100
GPNAM[4] SINE.100
GPNAM[5] SINE.100
GPZ1 80.00 %
GPZ3 15.00 %
GPZ4 -10.00 %
GPZ5 -5.00 %
P16 1000.00 usec

F1 - Acquisition parameters
TD 256
SFO1 150.9179 MHz
FIDRES 131.082214 Hz
SW 222.353 ppm
FnMODE Echo-Antiecho

F2 - Processing parameters
SI 2048
SF 600.1300005 MHz
WDW SINE
SSB 0
LB 0 Hz
GB 0
PC 1.40

F1 - Processing parameters
SI 512
MC2 echo-antiecho
SF 150.9025274 MHz
WDW SINE
SSB 2
LB 0 Hz
GB 0
