# Supplementary figures and images for: Neurotherapeutic effects of Ginkgo biloba extract and its terpene trilactone, ginkgolide B, on sciatic crush injury model: A new evidence
Source: PLoS One. 2019 Dec 26;14(12):e0226626. doi: 10.1371/journal.pone.0226626 (PMC6932810; doi:10.1371/journal.pone.0226626)

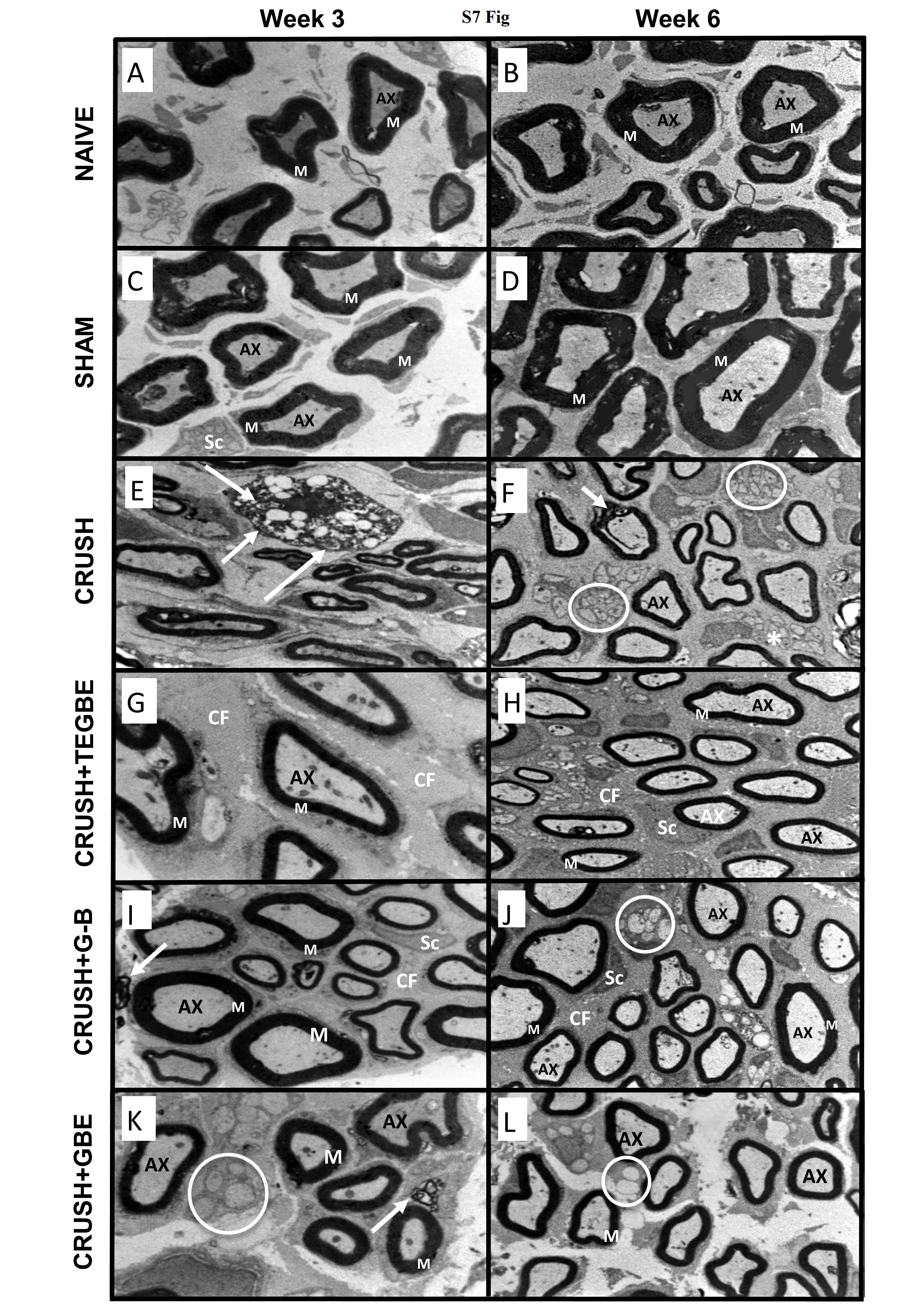

Supplement: S7 Fig — A: Sciatic nerve electron micrographs from the different experimental groups at week 3 and week 6 post-injury (5000x). Naïve (n = 6) (A and B) and sham (n = 12) (C and D) animals show the normal nerve fibers and healthy appearance of myelin sheaths (M) and axons (AX) at week 3 and week 6, respectively. The crush (E) group (n = 12), at week 3, exhibits very irregular shaped myelin sheaths and axonal fibers along with disintegrated and remnants of myelin scattered in between the axons (arrows). At week 6, the majority of the axons and nerve fibers in the crush group (F) are still small in size and thinly myelinated. Also, the crush nerves exhibit newly regenerated nerve fibers (circles) along with unmyelinated axonal fibers and disintegrated myelin sheaths (small arrow). The TEGBE (n = 12) (G and H), G-B (n = 12) (I and J) and GBE-treated (n = 6) (K and L) groups show normal nerve fibers with healthy appearance myelin sheaths and collagen fibers (CF) compared to (D and F). Note that at week 6 the all treated groups (H, J and L) displayed more remarkable intact and organized extracellular matrix in addition to normal and healthy myelin sheaths in addition to newly regenerated axonal fibers (circles). (TIF) [file pone.0226626.s007.tif]
